# Slaying little dragons: the impact of the Guinea Worm Eradication Program on dracunculiasis disability averted from 1990 to 2016

**DOI:** 10.12688/gatesopenres.12827.1

**Published:** 2018-06-18

**Authors:** Elizabeth A. Cromwell, Sharon Roy, Dieudonne P. Sankara, Adam Weiss, Jeffrey Stanaway, Ellen Goldberg, David M. Pigott, Heidi Larson, Stein Emil Vollset, Kristopher Krohn, Kyle Foreman, Peter Hotez, Zulfiqar Bhutta, Bayu Begashaw Bekele, Dumessa Edessa, Nicholas Kassembaum, Ali Mokdad, Christopher J. L. Murray, Simon I. Hay

**Affiliations:** 1 Institute for Health Metrics and Evaluation, University of Washington, Seattle, Seattle, WA, USA; 2 Centers for Disease Controls and Prevention, Atlanta, GA, USA; 3 World Health Organization, Geneva, Switzerland; 4 The Carter Center, Atlanta, GA, USA; 5 College of Medicine, Baylor University, Houston, TX, USA; 6 Aga Khan University, Karachi, Pakistan; 7 University of Gondar, Gondar, Ethiopia; 8 Haramaya Univerisity, Dire Dawa, Ethiopia; 9 Big Data Institute, Li Ka Shing Centre for Health Information and Discovery, University of Oxford, Oxford, UK

**Keywords:** Guinea Worm, dracunculiasis, disability adjusted life years, DALYs, eradication, prevalence, GBD, Global burden of disease, neglected tropical diseases, NTDs

## Abstract

**Background:** The objective of this study was to document the worldwide decline of dracunculiasis (Guinea worm disease, GWD) burden, expressed as disability-adjusted life years (DALYs), from 1990 to 2016, as estimated in the Global Burden of Disease study 2016 (GBD 2016). While the annual number of cases of GWD have been consistently reported by WHO since the 1990s, the burden of disability due to GWD has not previously been quantified in GBD.

**Methods:** The incidence of GWD was modeled for each endemic country using annual national case reports. A literature search was conducted to characterize the presentation of GWD, translate the clinical symptoms into health sequelae, and then assign an average duration to the infection. Prevalence measures by sequelae were multiplied by disability weights to estimate DALYs.

**Results:** The total DALYs attributed to GWD across all endemic countries (n=21) in 1990 was 50,725 (95% UI: 35,265–69,197) and decreased to 0.9 (95% UI: 0.5–1.4) in 2016. A cumulative total of 12,900 DALYs were attributable to GWD from 1990 to 2016.

**Conclusions:** Using 1990 estimates of burden propagated forward, this analysis suggests that between 990,000 to 1.9 million DALYs have been averted as a result of the eradication program over the past 27 years.

## Introduction

Dracunculiasis, also known as Guinea worm disease (GWD), is caused by the parasitic worm *Dracunculus medinensis* (literally, little dragon from Medina) ^1^. The transmission cycle begins when Guinea worm larvae are released into common stagnant sources of surface drinking water (e.g., ponds, lakes, unprotected shallow wells) where they are consumed by minute aquatic crustaceans (copepods). In about two weeks, the larvae inside the copepods develop into the infective stage ^2^. At this time, if contaminated water is ingested, larvae migrate through the intestinal wall into the connective tissues, where they mature and mate. Approximately 10–14 months post-infection, a painful, burning blister is created on the skin. The skin over the blister sloughs-off in about 48 hours, revealing the skin lesion and the anterior end of the worm. To relieve the pain, infected persons immerse the affected body part in water, triggering the worm to emerge through the skin and expel her larvae and the cycle begins again ^2^,^5^.

The subsequent ulcer is painful and can often become infected, but most individuals recover over a period of weeks to months. Permanent disability, as well as death, have been documented but are very rare ^6^. There is no specific chemotherapy for GWD nor vaccine available. Diagnosis occurs at emergence ^2^ and treatment is limited to case management to avoid secondary bacterial infections. In the context of eradication programs, the traditional practice of wrapping the worm around a stick as it slowly emerges has been replaced by wrapping the worm around a sterile gauze and is augmented with wound management using sterile bandages, topical antibiotic ointment, and treatment with anti-inflammatories ^7^.

The global campaign to eradicate Guinea worm began in 1980 at the U.S. Centers for Disease Control and Prevention (CDC) ^1^. The campaign gained momentum when Guinea worm eradication was proposed to measure the success of the International Drinking Water Supply and Sanitation Decade of 1981–1990 ^2^,^8^. The global eradication effort is led by national governments and communities, with the support of a coalition of partners including The Carter Center, WHO, CDC, UNICEF and other partners and donors ^8^. To break the cycle of transmission, national Guinea Worm Eradication Programs implement case detection and containment, provision of safe water sources, distribution of filter cloths and pipe filters, water source treatment with a larvicide (temephos), and health education ^7^.

In 1990, a total of approximately 624,000 cases were reported globally; in 2016, only 25 cases were reported across four remaining endemic countries ^9^. If the global campaign is successful, Guinea worm could be the second human disease in history eradicated by direct public health interventions ^10^. The costly up-front investment in eradication is often cited as a mechanism to avoid the repeated and ongoing costs of treatment and prevention ^11^,^12^. Although there is no consensus on the ideal methodology to quantify the economic or social benefits of eradication ^13^, the impact of eradication in terms of alleviating human suffering is clear ^12^.

The first report of GWD surveillance was released in 1982 ^14^ and the World Health Assembly resolution WHA39.21 was endorsed in 1986. The annual number of cases of GWD has been consistently reported by WHO since the 1990s, but the burden of disability due to GWD has not previously been quantified in the Global Burden of Diseases, Injuries, and Risk Factors Study (GBD). Several village-level studies conducted between 1970 and the mid-1990s have described the clinical presentation of GWD ^15^, and have quantified economic and productivity losses due to temporary and long-term disability. Given the historic accomplishments of global eradication efforts ^1^, as well as the impetus for continued investment, an estimate of the burden of GWD comparable to other diseases generated by the GBD 2016 framework is valuable. Disability-adjusted life years (DALYs) serve as a measure of overall disease burden, expressed as the number of years lost due to disability or early death. DALYs estimated by GBD are comparable between countries and through time; for example, the burden of GWD can be compared not only to other infectious diseases but chronic conditions as well ^16^. Further, comparison of estimates of burden from the early 1990s could be used to construct alternative scenarios of the burden of GWD had the eradication program had not been implemented. Each disease eradication event (this may be only the second) provides important data on the economic case for eradication. Moreover, because simply documenting contemporary DALYs ^16^ can lead to the misplaced interpretation that the case for continued investment in a disease is diminished, measuring the benefits of eradication over the entire period of intervention needs a longer-term perspective ^11^,^13^,^16^. Here we estimate DALYs attributable to GWD between 1990 and 2016 as part of GBD 2016.

## Methods

### The Global Burden of Disease study

The burden of GWD was estimated for the first time in GBD 2016 ^16^. GBD produces the only comprehensive DALYs for 333 diseases and injuries, from 1990 to the present, for 195 countries and territories ^16^. A detailed description of GBD 2016 methodology is presented elsewhere ^17^,^18^. Once the prevalence of a cause is estimated, data on severity and the occurrence of particular consequences of disease, or “sequelae,” are used to determine the proportion of prevalent cases experiencing each sequela. The sequelae are then matched to health states and assigned disability weights ^18^. GBD causes are collectively exhaustive and mutually exclusive.

***Data sources***. We extracted annual country-level Guinea worm case data from the Weekly Epidemiological Record (WER), published by WHO, as detailed in Supplementary File 1. In the early 1990s, there are years for which annual case data are either missing or inconsistent with preceding/following annual reports. Thus, annual case data were reviewed for completeness and plausibility by comparison with each country’s longitudinal case data and implausible case reports were excluded ( Supplementary File 1). For example, in the case of Niger, which reported 32,829 cases in 1991 and then 500 in 1992, followed by 25,346 in 1993, the 1992 case data were treated as an outlier. A literature search using the PubMed database identified peer-reviewed publications (see Table 1) that described the clinical presentation of GWD in terms of symptoms, sequelae, and duration of morbidity among individuals with incident or prevalent GWD.

**Table 1.** Summary of Guinea-worm-related disability data reported in peer-reviewed literature.

Country	Year(s)	Number of communities (sample size *)	Type of morbidity reported **	Mean duration	Proportion affected
Nigeria ^19^	1985	1 village (444 individuals)	Incapacitation	26 days	93.4%
Nigeria ^20^	1986	1 village (325 individuals)	Incapacitation	60 days	63%
Nigeria ^21^	1983–1984	295 households	Pain	12.7 weeks	
			Incapacitation	4.2 weeks	Severe: 58%
Nigeria ^22^	1993	2 villages (982 individuals)	Incapacitation	-	21%
Nigeria ^23^	1971–1975	17 villages (sample size not reported)	Pain	--	Severe: 12% Moderate: 31% Mild: 57%
			Incapacitation	100 days	--
			Infection at wound site	--	9.8%
			Musculoskeletal problems	--	
Nigeria ^15^	1971–1974	47 villages (563 individuals)	Pain	4.2 weeks to 7.2 weeks	--
			Infection at wound site	10 weeks	17.4%
			Musculoskeletal problems	-	4.6%
Uganda ^24^	1992	43 clusters (301 women surveyed)	Incapacitation	6 months	40%
Benin ^25^	1987–1989	2 villages (30 households)	Incapacitation	39–59 days (across 2 sites)	--
Ghana ^6^	1991	10 villages (195 individuals)	Pain	12–18 months	28.2%
			Incapacitation	--	34%
Ghana ^26^	1973	8 villages (20 men)	Incapacitation	2.4 to 5.3 weeks	90%
			Infection at wound site	--	45%

*Sample size represents total number of individuals with Guinea worm disease (either prevalent or historical cases). **The term “incapacitation” captures any reports of limited mobility or inability to perform daily tasks, as measurement of incapacitation was variable across studies. “Musculoskeletal problems” encompasses a wide range of complications affecting joints and tissues, arthritis, and complications due to infections that could affect the lower limb.

***Statistical analysis***. The incidence of GWD was then modeled individually for each country considered endemic in 1990 using Stata (Release 13; StataCorp; College Station, TX). Incidence was modeled using either reported case data (where available and plausible; see Supplementary File 1) or using a Poisson regression over time, by country. For years and locations for which case data were reported and considered plausible, 1,000 draws estimating incidence were generated using a beta distribution of cases and annual national population minus cases to introduce uncertainty, relying on the assumption that national Guinea Worm Eradication Program data reflect the annual case burden. As Guinea worm case data were assumed to represent incidence of new infections, we employed a Poisson regression *per country* to predict cases for years and locations for which case data were missing or excluded, with year as the predictor, and the national population as the offset. The predicted incidence and standard error from the Poisson model were then used to generate a random distribution of 1,000 incidence draws for those years with missing or incomplete case data. For comparison, the analysis was repeated entirely without exclusion of possible implausible data.

***Calculation of disability-adjusted life years (DALYs)***. To convert estimates of prevalence into quantifiable and comparable measures of disability, GBD first generates estimates of years lived with disability (YLDs), which for GWD are the product of prevalence and a disability weight for all sequelae, corrected for comorbidity. Disability weights are measured on a scale from 0 to 1, with 0 implying a state that is equivalent to full health and 1 a state equivalent to death. In order to generate an estimate of total DALYs from 1990 to 2016, cause-specific years lived with disability are summed with YLDs for each location and year.

For GWD, the following steps were taken to assign health states to sequelae. First, the results of the literature review of Guinea worm-related disability were used to identify sequelae related to Guinea worm emergence. For each sequela, an estimate of its duration (as a fraction of one year) was multiplied across the 1,000 incidence draws to approximate prevalence. A simulation was run to adjust disability weights for comorbidities for all causes of the same sequelae across GBD ^18^. For each sequela, the adjusted disability weights were multiplied by prevalence and summed to estimate DALYs for GWD by location and year for each draw. From the 1,000 draws we calculated the point estimate as the mean of the draws, and the 2.5 and 97.5 percentile draws used to construct 95% uncertainty intervals (UI) for the estimation years of 1990, 1995, 2000, 2005, 2010, and 2016, and then interpolated to create the entire 1990–2016 time series. The mean of these annual estimates were summed to produce a total number of DALYs attributable to GWD for 1990–2016.

To estimate the number of DALYs that could have occurred in the absence of eradication interventions four simple scenarios were explored. First, the estimate of total DALYs was extrapolated from 1990 to 2016 accounting for no other changes. Second, DALYs from 1990 were extrapolated according to the annual percentage change in country-level population growth using GBD population estimates. A third scenario assumed declines in DALYs occurred solely due to increased access to improved water sources using the GBD 2016 national water coverage covariate estimates from 1990–2016. In this scenario, the 1990 DALY estimate was reduced on an annual basis according to the increase in the proportion of individuals with access to improved water sources. Finally, to account for burden in the 1980s that GBD results do not capture, the 1986 estimate of 3.5 million annual ^4^ cases was held constant (of which 3.3 million cases estimated for Africa ^27^) and multiplied by the mean DALY per case from GBD. The total number of DALYs predicted via GBD 2016 were then subtracted from the total produced under each alternative scenario to estimate the number of DALYs averted due to the eradication campaign. For the 1986–2016 comparison, the burden of GWD was assumed to decline 20% annually from 1987–1989 as case data from this period are limited and not nationally representative for most locations.

## Results

### Summary of data sources

**Figure 1.**
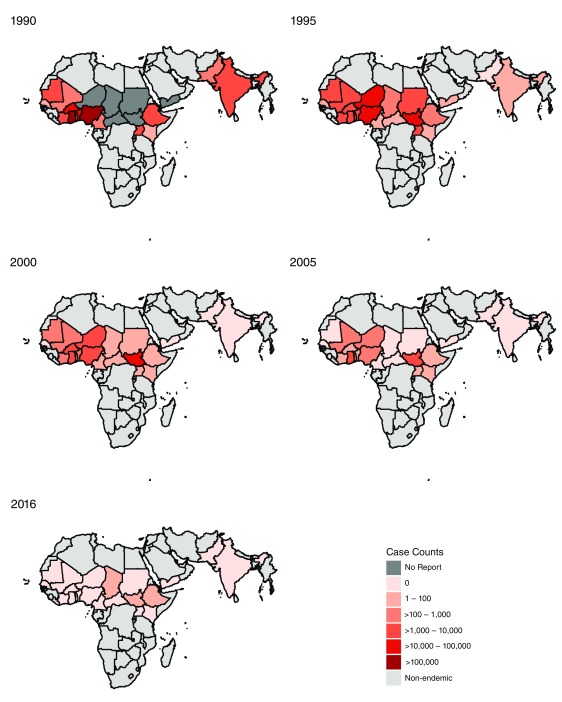
Distribution of Guinea worm disease: 1990, 1995, 2000, 2005, 2010, and 2016. Distribution of GWD cases for 1990, 1995, 2000, 2005, 2010, and 2016. In dark grey, countries with ongoing transmission but no report; in light grey, countries never considered endemic; and in shades of red total annual case counts, including countries with interruption of transmission that had imported cases.

A total of 21 countries were considered Guinea worm-endemic as of 1990 ( Figure 1) ^28^. Sudan and South Sudan were modelled separately in GBD for the entire time series even though they were one country until 2011. A total of 729 country-year-specific data points were identified ( Supplementary File 1). The literature review identified ten papers in which Guinea-worm-related morbidity was reported, summarized in Table 1. Sequelae associated with GWD relate to the process of the worm’s emergence: pain and itch as the worm exits the body, and the subsequent wound that requires several weeks to heal and which can be further complicated by abscesses and chronic ulcerations, joint and tissue damage, as well as secondary infection in connective tissues ^29^. Pain and itch were widely reported during the worm’s emergence; these persist for approximately one month until the worm exits the body ^2^. Several studies ^20^,^23^,^24^,^30^,^31^ found that worms predominantly emerge from the lower limbs, with reports ranging from 98% ^22^ to 88% ^15^ of all cases.

We assumed that every case of GWD, using GBD health state terminology, experienced “pain and disfigurement (moderate),” and “musculoskeletal problems, lower limb (moderate)” for a period of one month, followed by two months of “pain and disfigurement (mild).” Based on evidence from a study of long-term disability conducted in Ghana ^6^, we then assumed that 30% of all case-patients then experienced “pain and disfigurement (mild)” for an additional nine months (approximately a total year of disability) to account for longer-term disability associated with recovery. The disability weights ^32^ associated with these sequelae are as follows: moderate disfigurement (with itch/pain), 0.188, 95% UI: 0.125–0.267; musculoskeletal problems, lower limbs (moderate), 0.079, 95% UI: 0.054–0.11; and mild disfigurement (with itch/pain), 0.027, 95% UI: 0.015–0.042.

### Results of national-level GWD incidence estimates

Table 2 presents the total number of reported cases, modeled cases, total DALYs, and DALYs per capita for 1990, as well as the total country-specific DALYs for 2016 for comparison (individual country models are presented in Supplementary File 1). Overall, the GBD model predicts a total of approximately 1.6 million cases of GWD in 1990 and 27 cases in 2016 (compared to 25 cases reported in 2016). A total of 13 countries were missing case reports for at least one year, and a total of 18 country-years of data were considered implausible (see Supplementary File 1), accounting for the large difference in reported and modeled case burden, particularly in 1990 and 1995. Had these possible outliers not been excluded, the model would have predicted approximately 1.1 million cases of GWD for 1990.

**Table 2.** Country-specific burden estimates, comparing 1990 to 2016.

	1990								2016
Country	Reported Cases	Predicted Cases †	Total DALYs			DALYs per capita			DALYs Total
			Point Estimate	Lower UI	Upper UI	Point Estimate	Lower UI	Upper UI	
Benin	37,414	37,410.5	1,139.6	783.8	1,563.8	23.2	15.9	31.8	-
Burkina Faso	42,187	42,190.1	1,277.2	874.9	1,753.4	14.6	9.9	20.0	-
Cameroon	742	742.1	24.2	16.0	34.0	0.2	0.1	0.3	-
Central African Republic	*	188.5	6.2	3.4	10.1	0.2	0.1	0.3	-
Chad	*	4,912.1	151.2	101.6	215.2	2.6	1.7	3.7	0.5
Côte d’Ivoire	1,360	21,323.4	652.2	436.8	897.3	5.4	3.6	7.5	-
Ethiopia	2,333	2,332.7	76.1	51.5	106.4	0.2	0.1	0.2	0.09
Ghana	123,793	123,810.0	3,804.7	2,616.5	5,210.4	26.0	18.0	35.7	-
India	4,798	4,800.3	156.4	105.9	217.9	0.02	0.01	0.03	-
Kenya	6	61.4	2.0	1.2	3.1	0.01	0.01	0.01	-
Mali	884	21,723.36	660.0	454.2	906.1	7.8	5.4	10.1	0.0
Mauritania	8,036	8,034.3	246.2	167.4	339.0	12.5	8.5	17.2	-
Niger	*	61,698.8	1,896.3	1,280.2	2,618.4	24.3	16.4	33.6	
Nigeria	394,082	394,078.7	12,067.6	8,345.6	16,593.6	12.8	8.9	17.7	-
Pakistan	160	160.1	5.2	3.4	7.5	0.004	0.003	0.01	-
Senegal	38	2,987.8	96.0	64.6	133.9	1.3	0.9	1.8	-
South Sudan **	*	229,724.3	6,939.9	4,811.8	9,394.0	115.1	79.8	155.8	0.2
Sudan	*	8,116.9	260.7	174.1	363.8	1.4	1.0	1.9	-
Togo	3,042	16,659.15	510.2	344.7	704.5	13.7	9.2	18.9	-
Uganda	4,704	678,265.5	20,656.7	14,294.5	28,233.3	117.7	82.5	160.9	-
Yemen	*	2,562.8	82.1	43.2	141.1	0.7	0.4	1.2	-
**Total**	**623,579**	**1,661,782.7**	**50,725.4**	**35,265.3**	**69,197.0**	-	-	-	**0.9**

DALYs - Disability-adjusted life years **†**Mean prediction from 1,000 draws. *No data reported for 1990. **South Sudan: Data for Sudan and South Sudan were disaggregated according to current national boundaries for the entire period 1990–2016 as GBD estimates are generated for current political boundaries. We acknowledge that pre-2011, Sudan and South Sudan were not separate countries and that pre-2006 Guinea worm eradication was implemented as a single national program ^33^.

### DALY estimates

The total DALYs attributed to GWD across all endemic countries in 1990 was 50,725 (95% UI: 35,300–69,200). From 1995 onward, the DALYs attributable to GWD dropped precipitously, with 1995 DALY estimates of 4,020 (95% UI: 2,750–5,530), consistent with the expansion of eradication efforts in Africa throughout the mid-1990s and 2000s. In 1990, the majority of the DALYs occurred in Uganda (20,700; 95% UI: 14,300–28,200), Nigeria (12,100; 95% UI: 8,300–16,600), and what is now South Sudan (6,940; 95% UI: 4,810–9,390). In terms of DALYs per 100,000, estimates in 1990 were the highest in Uganda (118; 95% UI: 82–161), South Sudan (115; 95% UI: 80–156), and Ghana (26; 95% UI: 18–36)). For comparison against other causes, the 1990 DALY estimates per 100,000 indicate relative burden. For example, in Ghana, the 1990 DALY estimate for lymphatic filariasis was 80 (95% UI: 40-135) and schistosomiasis was 276 (95% UI: 182—435). In Nigeria, the DALYs for lymphatic filariasis and schistosomiasis in 1990 were 208 (95% UI: 95—377) and 301 (185—491), respectively (see GHDx search tool to search results by country and year).

By interpolating the quinquennial DALY estimates, the total cumulative DALYs due to GWD from 1990 to 2016 was approximately 129,355 (sum of the mean model prediction). In the first scenario we tested in which the estimate of total DALYs from 1990 (50,725.4 DALYs per year) is held constant through 2016, a cumulative 1.37 million DALYs could have occurred in the absence of any change in GWD incidence. In the second scenario in which the 1990 DALY estimate is extrapolated according to annual population growth, approximately 2 million DALYs could have occurred. Third, accounting for secular improvements in improved water source access we predict a total of 1.1 million DALYs. Using these three scenarios, the number of DALYs averted by the Guinea worm eradication campaign may be as low as 990,000 or as high as 1.89 million (see Table 3). Finally, by multiplying the mean DALYs per case generated by GBD (approximately 0.03) by the 1986 global case estimate of 3.5 million ^4^, we project a total of 106,836 DALYs per year for a total of 3.3 million DALYs that could have occurred from 1986–2016 had the 1986 burden remained constant. In this scenario, we calculated 444,736 DALYs occurred under the eradication campaign, allowing for annual case reductions of 20% from 1987–1989 and GBD 2016 results for the period 1990–2016, resulting in approximately 2.8 million DALYs averted from 1986–2016.

**Table 3.** Global Guinea worm disease disability-adjusted life years (DALYs) averted comparing observed and alternative scenarios.

Inputs	DALYs
Estimated cases *	1,661,783
DALYs 1990 *	50,725
DALYs observed 1990–2016 *	129,355
Annual 1990 burden remains constant	1,369,586
Increases with population growth	2,014,473
Changes in proportion with safe water coverage	1,119,703
DALYs averted: constant burden	1,240,231
DALYs averted: increases with population	1,885,118
DALYs averted: changes in safe water	990,348

*Using Global Burden of Disease 2016 results.

## Discussion

This study summarizes the first model of GWD included in the GBD study, estimating the burden of disease attributable to Guinea worm from 1990 to 2016. In comparison to GBD results, previously published studies present similar estimates of case burden, particularly for the early 1990s. The first model published focused on incidence ^27^ using reported case data corrected for underreporting, estimating approximately 3.3 million incident cases of GWD in Africa occurred in 1986 across 19 countries (Sudan and South Sudan modeled as one country); including India and Pakistan the estimate is 3.5 million cases for 1986 ^4^. Another analysis, conducted in collaboration with the World Bank, estimated approximately 1.5 million cases in 1990 ^34^, a number similar to the 1990 results herein. Other studies have presented the contribution of Guinea worm eradication in the context of Millennium Development Goals ^35^ and economic productivity gains ^34^, and a recent economic analysis calculates DALYs averted per dollar as a metric to justify economic investment in eradication versus control ^36^. Here we similarly focus on DALYs averted as estimated by GBD 2016, with country-level DALYs presented for the first time.

Whether disease eradication is motivated by an economic and/or moral imperative, there are challenges inherent in quantifying the benefits attained. The DALY is a metric that enables decision-makers and public health officials to compare across different causes based on the disease experience, not only the decline in cases. Our analysis includes a review of the scientific literature on GWD, which is sparse and largely published before 1990. These studies may not be representative of the morbidity experienced during the eradication campaign as case management interventions reached scale. Since we also did not account for the quantity of worm burden per case, our current DALY estimates may underestimate the true burden of GWD, as evidence suggests severity of disability is related to the number of worms ^23^. Secondary complications associated with worm emergence that persisted beyond a year post-emergence were not included, as data on the long-term clinical outcomes of GWD were scarce. Assumptions used in other analyses are based on single studies with very small numbers ^36^. For example, the estimate of 0.5% ^6^ of all cases resulting in permanent disability results from a report of one individual with permanent damage to a joint post worm emergence from a sample of 195 individuals. Given the intensity of community-based surveillance over the course of the eradication campaign, it might be possible to generate better parameters for GWD sequelae if detailed case data could be made available.

Our analysis is the first to quantify the DALY burden of GWD in the GBD 2016 framework and is subject to all GBD 2016-specific limitations ^16^,^18^,^37^. First, due to the scope of GBD, we were only able to analyze from 1990 onward. It is plausible that case data in the early 1990s do not capture the true incidence of GWD in Africa due to under-reporting as many national eradication programs had not reached full geographic coverage of endemic areas. In terms of historical burden, this analysis also does not account for the cases that were reported prior to 1990 from India and Pakistan, countries which had begun eradication efforts in 1980 and 1987 ^38^, respectively, much earlier than most of the other countries (see SI for summary of national case searches). Had our analysis accounted for a larger proportion of the historical burden the number of DALYs averted by eradication campaign interventions would be greater. Second, we attempted to correct for under-reporting by omitting implausible annual country reports which also could have introduced bias into the 1990 estimate. Nevertheless, to ignore missing or implausible data points would have otherwise introduced clear downward bias in our DALY estimates, understating the true burden during this period. Prior evidence acknowledges incomplete reporting ^39^, and our analysis introduced greater levels of uncertainty for years in which case estimates were missing or considered implausible. If data points that were considered outliers had not been excluded from the analysis, the total number of estimated cases would be 1.1 million, still far greater than the 623,579 cases reported by WHO in 1990. This increase would account for missing case data from Central African Republic, Chad, Niger, South Sudan, Sudan and Yemen. Further, reliance on nationally representative data did not enable us to account for the subnational distribution of disease, which may have resulted in over-prediction for country-years that were missing data, notably for Uganda and South Sudan.

Our model suggests that approximately 129,000 DALYs were attributable to GWD for the entire period 1990–2016. As India and Pakistan began national eradication efforts much earlier, this burden largely occurred in Africa, with approximately 50,000 DALYs estimated in 1990 alone. A simple extrapolation of the 1990 mean DALY estimate would imply that 1.3 million DALYs could have occurred in the absence of the eradication program from 1990 onward. Accounting for population growth, that estimate grows to 2.0 million. Using only national level measures of safe water quality to account for secular improvements that could have eliminated transmission, this would suggest that approximately 990,000 DALYs have been averted over the past 27 years. Although data availability pre-1990 limit our ability to construct complex alternative scenarios, the reduction in GWD case reports outpaced national-level measures of water quality. There was approximately an 80% reduction in reported cases of GWD from 1990 to 1995, whereas the change in water access at the national level (a GBD covariate) during this period ranged from a decrease of 2% in coverage (Pakistan) to an increase of 23% (Sudan), suggesting the eradication campaign interventions such as filter cloths, health education and case containment account for a large proportion of the reduction in burden.

While these alternative scenarios present simple estimates of the possible GWD-related disability that has been prevented since 1990, future analyses with more detailed national-level data could generate better estimates. It is likely our estimates of DALYs averted under-estimate the true impact of eradication as interventions in other high-burden settings like Ghana and Nigeria were already underway. These estimates also do not capture declines in GWD incidence occurring in India ^40^ and Pakistan ^41^ in the 1980s, which combined reported between 20,000-30,000 cases per year combined. Using the 1986 estimate of 3.5 million cases ^4^ would double the burden of GWD that we estimated for 1990 and result in approximately 2.8 million DALYs averted (assuming 1986 burden held constant) from 1986–2016.

The future of Guinea worm eradication will depend on a number of factors, including elimination of infection in animals, surveillance in settings with insecurity, and maintenance of a programmatic infrastructure prior to elimination of transmission ^42^. Critics of eradication programs may claim that the “cost per case” to sustain interventions at this late stage could be better allocated to more pressing public health priorities. Nonetheless, a recent economic analysis shows that eradication is still effective even at this late stage ^36^. Should eradication efforts continue, the small number of cases reported will not change the overall results of historical DALYs estimated in this study, even if eradication takes decades. The costs expended versus DALYs saved calculations will look increasingly disproportionate as annual case totals continue to decline. By quantifying the contribution of the global eradication program in terms of DALYs averted, we demonstrate the huge benefit in the reduction of human suffering. This study is important as it facilitates a more holistic assessment of the entire achievement of the campaign while the final stubborn cases are eliminated.

## Disclaimer

The findings and conclusions in this report are those of the authors and do not necessarily represent the official position of the Centers for Disease Control and Prevention.

## Data availability

Data obtained using the Global Burden of Disease Exchange: http://ghdx.healthdata.org/

A full set of extracted data is available from Open Science Framework. Dataset 1: Guinea Worm dataset. http://doi.org/10.17605/OSF.IO/B63JD ^43^

This dataset is available under a CC0 1.0 Universal license.

## Software availability

Code used to generate estimates is available from Github: https://github.com/ihmeuw/ihme-modeling/tree/master/nonfatal_code/ntd_guineaworm

Archived source code at the time of publication is available from Zenodo: http://doi.org/10.5281/zenodo.1285962 ^44^

The code is available under a CC by 4.0 license

## Supplementary material

Supplementary File 1 – Guinea worm case data by country or subnational division with country specific models results.
