# Supplementary material for: Slaying little dragons: the impact of the Guinea Worm Eradication Program on dracunculiasis disability averted from 1990 to 2016
Source: Gates Open Res. 2018 Jun 18;2:30. [Version 1] doi: 10.12688/gatesopenres.12827.1 (PMC6139381; doi:10.12688/gatesopenres.12827.1)
Supplement: Supplementary file 1 [file gatesopenres-2-13900-s0000.tgz › e79d4ef7-2655-449e-9160-67246e0f743f.docx]

**Supplemental Information**

1. Background on Guinea worm case data^1-7^

The country-level annual case reports enumerate the number of individuals with Guinea worm disease in a calendar year (January-December). These individuals were typically counted as a case only once in a given year, when the first Guinea worm emerged; if that individual went on to experience subsequent cases in that calendar year those were not counted separately. To ensure that cases were attributed to the burden of Guinea worm disease in the country in which the case was detected, both indigenous and imported cases were included.

Case data in the early 1990s suggests under-reporting. To account for implausible case reports, the data were reviewed longitudinally. If there is an order of magnitude difference in the case series, the lower year was outliered from the main analysis. For example, Niger: 1991: 32,829 cases; 1992: 500 cases; 1993: 25,346 cases). Table 2 presents the year annual case searches were complete, as well as reporting rates from 1995 to justify exclusion of data points from the early 1990s.

By design, the Guinea worm eradication programmatic infrastructure covers the entire at-risk population in endemic countries. Since case containment is a key intervention designed to not only interrupt transmission but also monitor progress towards eradication, incident cases of guinea worm disease are generally considered nationally representative. Case reporting occurs at the village level on a monthly basis; case data are then aggregated within the national Guinea Worm Eradication Program and reported to the World Health Organization. In settings where annual case reports are low (suggesting no transmission) or transmission has been interrupted, cash rewards are promoted to enhance surveillance activities until national elimination is certified. Containment is defined as: detection within 24 hours of the worm's emergence; the patient did not contaminate any water source; the patient received proper wound care and health education on not entering any water source; a supervisor verified the case as dracunculiasis within seven days; and Abate® is used if there is any uncertainty about contamination of water sources or known contamination of water sources.

### SI Table 1: Guinea worm case data by country or subnational division, by year

| **Geography** | **1990** | **1991** | **1992** | **1993** | **1994** | **1995** | **1996** | **1997** | **1998** | **1999** |
| --- | --- | --- | --- | --- | --- | --- | --- | --- | --- | --- |
| Benin | 37417 | 4006* | 4315* | 16334 | 4302 | 2273 | 1427 | 855 | 695 | 492 |
| Burkina Faso | 42187 | * | 11784 | 8281 | 6861 | 6281 | 3241 | 2477 | 2227 | 2184 |
| Cameroon | 742 | 393 | 127 | 72 | 30 | 15 | 17 | 19 | 23 | 8 |
| Central African Republic | * | * | * | * | * | 18 | 9* | 5* | 34 | 26 |
| Chad | * | * | 156* | 1231 | 640 | 149 | 127 | 25 | 3 | 1 |
| Cote d’Ivoire | 1360* | 12690 | * | 8034 | 5061 | 3801 | 2794 | 1254 | 1414 | 476 |
| Ethiopia | 2333 | * | 303* | 1120 | 1252 | 514 | 371 | 451 | 366 | 249 |
| Ghana | 123793 | 66697 | 33464 | 17918 | 8432 | 8894 | 4877 | 8921 | 5473 | 9027 |
| India |  |  |  |  |  |  |  |  |  |  |
| Andhra Pradesh | 207 | 120 | 26 | 0 | 0 | 0 | 0 | 0 | 0 | 0 |
| Gujarat | 22 | 0 | 0 | 0 | 0 | 0 | 0 | 0 | 0 | 0 |
| Karnataka | 634 | 226 | 167 | 29 | 10 | 0 | 0 | 0 | 0 | 0 |
| Madhya Pradesh | 333 | 120 | 91 | 179 | 13 | 0 | 0 | 0 | 0 | 0 |
| Maharashtra | 209 | 0 | 1 | 0 | 0 | 0 | 0 | 0 | 0 | 0 |
| Rajasthan | 3376 | 1712 | 792 | 547 | 348 | 60 | 90 | 0 | 0 | 0 |
| Tamil Nadu | 0 | 1 | 0 | 0 | 0 | 0 | 0 | 0 | 0 | 0 |
| Telangana | 17 | 6 | 4 | 0 | 0 | 0 | 0 | 0 | 0 | 0 |
| Kenya |  |  |  |  |  |  |  |  |  |  |
| Turkana County | 6* | * | * | 35 | 37 | 23 | 0 | 6 | 7 | 1 |
| Mali | 884* | 16024 | * | 12011 | 5581 | 4218 | 2402 | 1099 | 650 | 410 |
| Mauritania | 8036 | * | 1557* | 5882 | 5029 | 1762 | 562 | 388 | 379 | 255 |
| Niger | * | 32829 | 500* | 25346 | 18562 | 13821 | 2956 | 3030 | 2700 | 1920 |
| Nigeria | 394082 | 281937 | 183169 | 75752 | 39774 | 16374 | 12282 | 12590 | 13420 | 13237 |
| Pakistan | 160 | 106 | 23 | 2 | 0 | 0 | 0 | 0 | 0 | 0 |
| Senegal | 38* | 1341 | 728 | 815 | 195 | 76 | 19 | 4 | 0 | 0 |
| South Sudan** | * | * | * | * | 53139 | 60555 | 116844* | 42944 | 47126 | 65805 |
| Sudan | * | * | 2447 | 2984 | 132* | 4053 | 1734 | 652 | 851 | 292 |
| Togo | 3042* | 5118* | 8179 | 10349 | 5044 | 2073 | 1626 | 1762 | 2128 | 1589 |
| Uganda | 4704* | * | 126369* | 42852 | 10425 | 4810 | 1455 | 1374 | 1061 | 321 |
| Yemen | * | * | * | * | 106 | 82 | 62 | 7 | 0 | 0 |

*****Years for which data were missing or considered implausible.

******Although South Sudan was not independent until 2011, the GBD estimates disease burden using current political boundaries and applies those boundaries retrospectively.

### SI Table 1 (continued): Guinea worm case data by country or subnational division, by year

| **Geography** | **2000** | **2001** | **2002** | **2003** | **2004** | **2005** | **2006** | **2007** | **2008** | **2009** |
| --- | --- | --- | --- | --- | --- | --- | --- | --- | --- | --- |
| Benin | 186 | 172 | 181 | 30 | 3 | 1 | 0 | 0 | 0 | 0 |
| Burkina Faso | 1956 | 1032 | 591 | 203 | 60 | 30 | 5 | 3 | 1 | 0 |
| Cameroon | 5 | 5 | 3 | 0 | 0 | 0 | 0 | 0 | 0 | 0 |
| Central African Republic | 35 | 36 | * | 0 | 0 | 0 | 0 | 0 | 0 | 0 |
| Chad | 3 | 0 | 0 | 0 | 0 | 0 | 0 | 0 | 0 | 0 |
| Cote d’Ivoire | 297 | 231 | 198 | 42 | 21 | 10 | 5 | 0 | 0 | 0 |
| Ethiopia | 60 | 29 | 47 | 28 | 17 | 37 | 3 | 3 | 41 | 24 |
| Ghana | 7402 | 4739 | 5611 | 8290 | 7275 | 3981 | 4136 | 3358 | 501 | 242 |
| India |  |  |  |  |  |  |  |  |  |  |
| Andhra Pradesh | 0 | 0 | 0 | 0 | 0 | 0 | 0 | 0 | 0 | 0 |
| Gujarat | 0 | 0 | 0 | 0 | 0 | 0 | 0 | 0 | 0 | 0 |
| Karnataka | 0 | 0 | 0 | 0 | 0 | 0 | 0 | 0 | 0 | 0 |
| Madhya Pradesh | 0 | 0 | 0 | 0 | 0 | 0 | 0 | 0 | 0 | 0 |
| Maharashtra | 0 | 0 | 0 | 0 | 0 | 0 | 0 | 0 | 0 | 0 |
| Rajasthan | 0 | 0 | 0 | 0 | 0 | 0 | 0 | 0 | 0 | 0 |
| Tamil Nadu | 0 | 0 | 0 | 0 | 0 | 0 | 0 | 0 | 0 | 0 |
| Telangana | 0 | 0 | 0 | 0 | 0 | 0 | 0 | 0 | 0 | 0 |
| Kenya |  |  |  |  |  |  |  |  |  |  |
| Turkana County | 4 | 8 | 17 | 12 | 7 | 2 | 0 | 0 | 0 | 0 |
| Mali | 290 | 718 | 861 | 829 | 357 | 659 | 329 | 313 | 417 | 186 |
| Mauritania | 136 | 94 | 42 | 13 | 3 | 0 | 0 | 0 | 0 | 0 |
| Niger | 1166 | 417 | 248 | 293 | 240 | 183 | 110 | 14 | 3 | 5 |
| Nigeria | 7869 | 5355 | 3820 | 1459 | 495 | 120 | 16 | 73 | 38 | 0 |
| Pakistan | 0 | 0 | 0 | 0 | 0 | 0 | 0 | 0 | 0 | 0 |
| Senegal | 0 | 1 | 0 | 0 | 0 | 0 | 0 | 0 | 0 | 0 |
| South Sudan** | 54800 | 49339 | 41403 | 20270 | 7255 | 5569 | 20582 | 5815 | 3618 | 2733 |
| Sudan | 90 | 132 | 90 | 29 | 11 | 0 | 0 | 0 | 0 | 0 |
| Togo | 1354 | 1502 | 669 | 278 | 73 | 29 | 2 | 0 | 0 | 0 |
| Uganda | 96 | 55 | 24 | 26 | 4 | 9 | 2 | 4 | 0 | 0 |
| Yemen | 0 | 1 | 0 | 0 | 0 | 0 | 0 | 0 | 0 | 0 |

*****Years for which data were missing or considered implausible.

******Although South Sudan was not independent until 2011, the GBD estimates disease burden using current political boundaries and applies those boundaries retrospectively.

### SI Table 1 (continued): Guinea worm case data by country or subnational division, by year

| **Geography** | **2010** | **2011** | **2012** | **2013** | **2014** | **2015** | **2016** |
| --- | --- | --- | --- | --- | --- | --- | --- |
| Benin | 0 | 0 | 0 | 0 | 0 | 0 | 0 |
| Burkina Faso | 0 | 0 | 0 | 0 | 0 | 0 | 0 |
| Cameroon | 0 | 0 | 0 | 0 | 0 | 0 | 0 |
| Central African Republic | 0 | 0 | 0 | 0 | 0 | 0 | 0 |
| Chad | 10 | 10 | 10 | 14 | 13 | 9 | 16 |
| Cote d’Ivoire | 0 | 0 | 0 | 0 | 0 | 0 | 0 |
| Ethiopia | 21 | 8 | 4 | 7 | 3 | 3 | 3 |
| Ghana | 8 | 0 | 0 | 0 | 0 | 0 | 0 |
| India |  |  |  |  |  |  |  |
| Andhra Pradesh | 0 | 0 | 0 | 0 | 0 | 0 | 0 |
| Gujarat | 0 | 0 | 0 | 0 | 0 | 0 | 0 |
| Karnataka | 0 | 0 | 0 | 0 | 0 | 0 | 0 |
| Madhya Pradesh | 0 | 0 | 0 | 0 | 0 | 0 | 0 |
| Maharashtra | 0 | 0 | 0 | 0 | 0 | 0 | 0 |
| Rajasthan | 0 | 0 | 0 | 0 | 0 | 0 | 0 |
| Tamil Nadu | 0 | 0 | 0 | 0 | 0 | 0 | 0 |
| Telangana | 0 | 0 | 0 | 0 | 0 | 0 | 0 |
| Kenya |  |  |  |  |  |  |  |
| Turkana County | 0 | 0 | 0 | 0 | 0 | 0 | 0 |
| Mali | 57 | 12 | 4 | 11 | 40 | 5 | 0 |
| Mauritania | 0 | 0 | 0 | 0 | 0 | 0 | 0 |
| Niger | 3 | 0 | 3 | 0 | 0 | 0 | 0 |
| Nigeria | 0 | 0 | 0 | 0 | 0 | 0 | 0 |
| Pakistan | 0 | 0 | 0 | 0 | 0 | 0 | 0 |
| Senegal | 0 | 0 | 0 | 0 | 0 | 0 | 0 |
| South Sudan** | 1698 | 1028 | 521 | 113 | 70 | 5 | 6 |
| Sudan | 0 | 0 | 0 | 3 | 0 | 0 | 0 |
| Togo | 0 | 0 | 0 | 0 | 0 | 0 | 0 |
| Uganda | 0 | 0 | 0 | 0 | 0 | 0 | 0 |
| Yemen | 0 | 0 | 0 | 0 | 0 | 0 | 0 |

*****Years for which data were missing or considered implausible.

******Although South Sudan was not independent until 2011, the GBD estimates disease burden using current political boundaries and applies those boundaries retrospectively.

1. **Summary of country-level Guinea worm case searches and reporting**

### SI Table 2: National case search completion and reporting rate

| **Country** | **National case search completed** | **Reporting rate 1995^8^ (%)** |
| --- | --- | --- |
| Benin | 1990^9^ | 81 |
| Burkina Faso | 1990^9^ | 80 |
| Cameroon | 1990^10^ | 86 |
| Central African Republic | 1991^9^ | NR* |
| Chad | 1994^11^ | 99 |
| Cote d’Ivoire | 1991^9^ | 95 |
| Ethiopia | 1993^12^ | 86 |
| Ghana | 1989^10^ | 88 |
| India | 1980^13^ | 100 |
| Kenya | 1994^14^ | NR* |
| Mali | 1992^15^ | 87 |
| Mauritania | 1991^9^ | 96 |
| Niger | 1991^9^ | 88 |
| Nigeria | 1990^16^ | 79 |
| Pakistan | 1987^13^ | - |
| Senegal | 1991^9^ | 100 |
| South Sudan** | - | NR* |
| Sudan | 1993^12^ | NR* |
| Togo | 1991^9^ | 94 |
| Uganda | 1992^17^ | 95 |
| Yemen | 1995^18^ | 99 |

*NR: Not reported.

**South Sudan and Sudan Guinea worm eradication program data were reported under one national program until 2006. Case searches and annual reporting in the 1990s was limited to accessible areas.

1. **Country-specific model results**

The countries with the starkest difference between reported cases and cases predicted by the model were Uganda and South Sudan, largely due to either the high case burden or sequential years of missing data. In the case of Uganda, the 1992 case data were set to missing because their inclusion resulted in over 1 million cases predicted in 1990, which upon review of preliminary results was considered a gross over-prediction. For South Sudan, inclusion of the 1996 data point of 116,844 cases also resulted in vast over-prediction of cases in the early 1990s. While we do not dispute the case burden in these two countries was likely quite high, in order to generate stable model estimates overall, these points were treated as outliers in the analysis. In contrast, Central African Republic (CAR) and Kenya model predictions did not fit the data well due to the extremely low numbers (<30 cases) as well as several years of missing data.


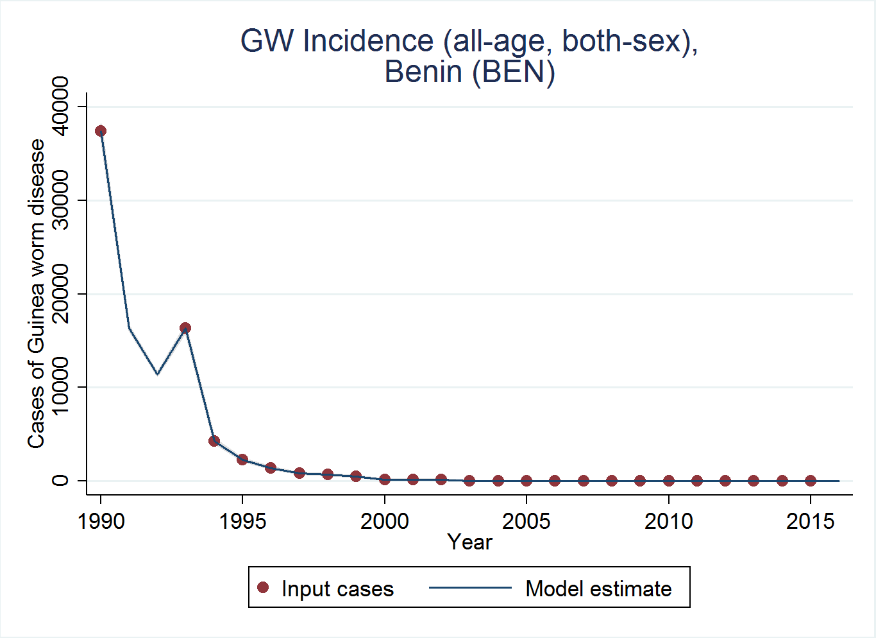

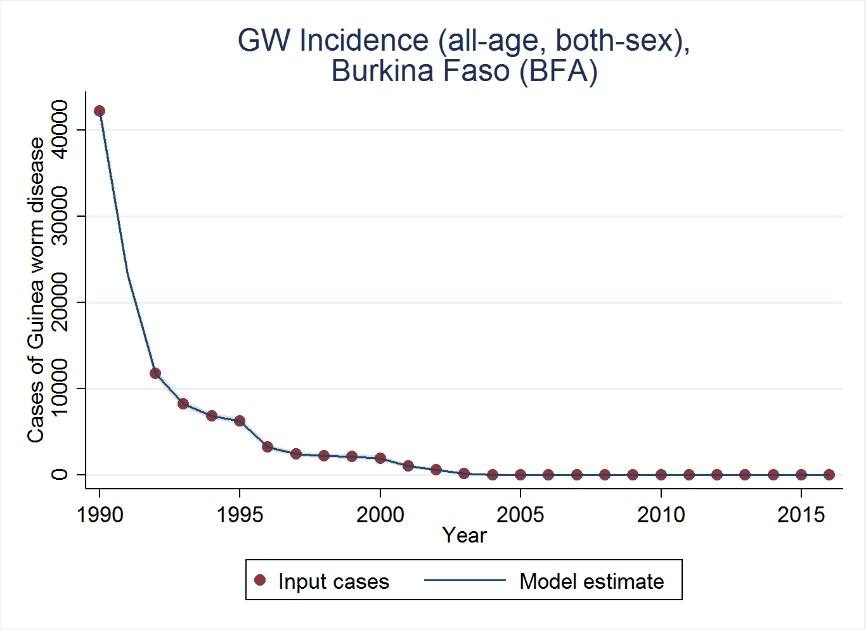


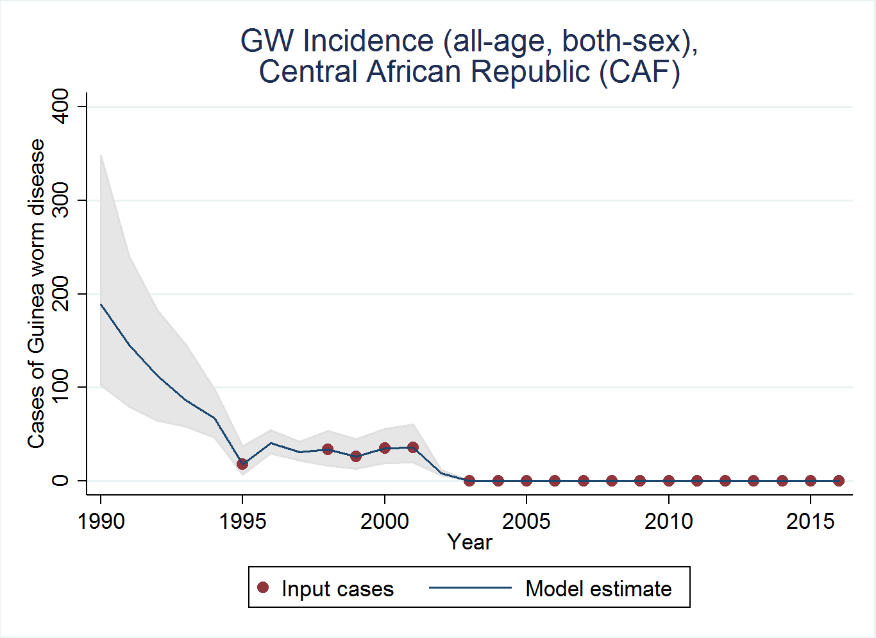

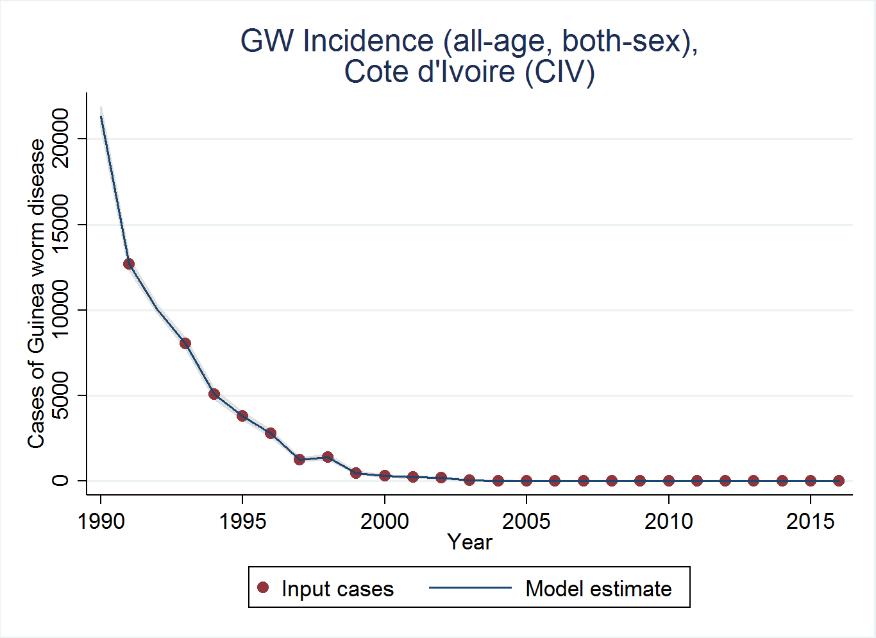


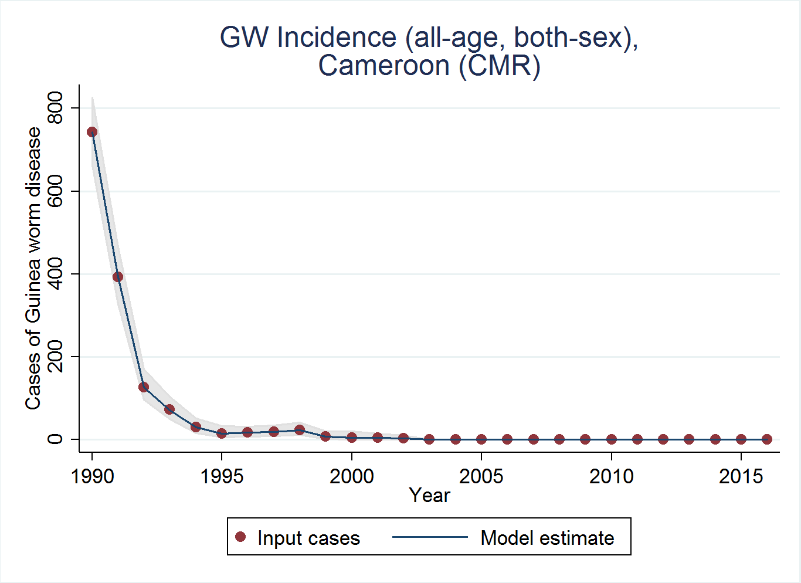

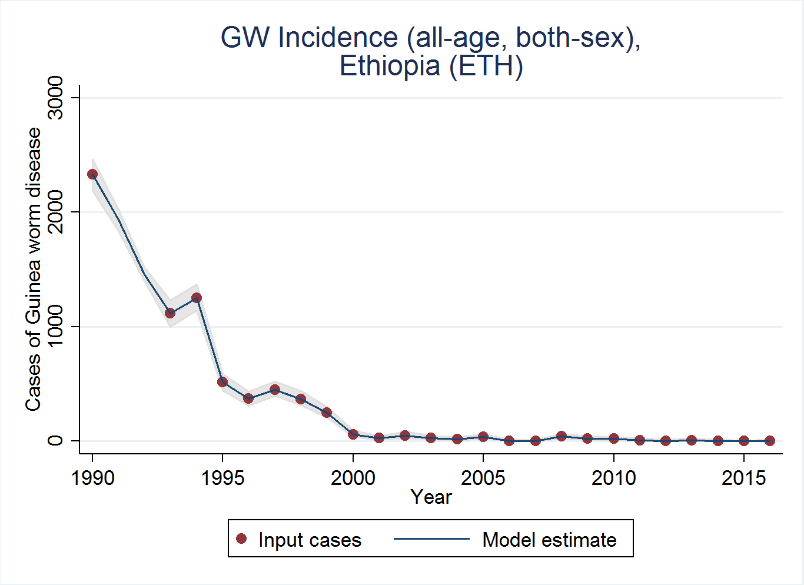


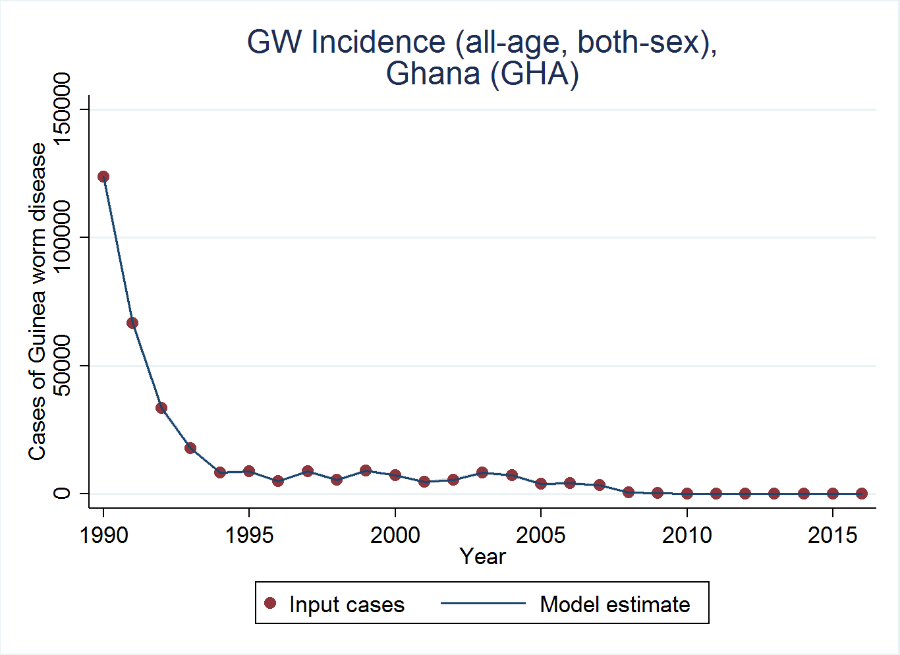

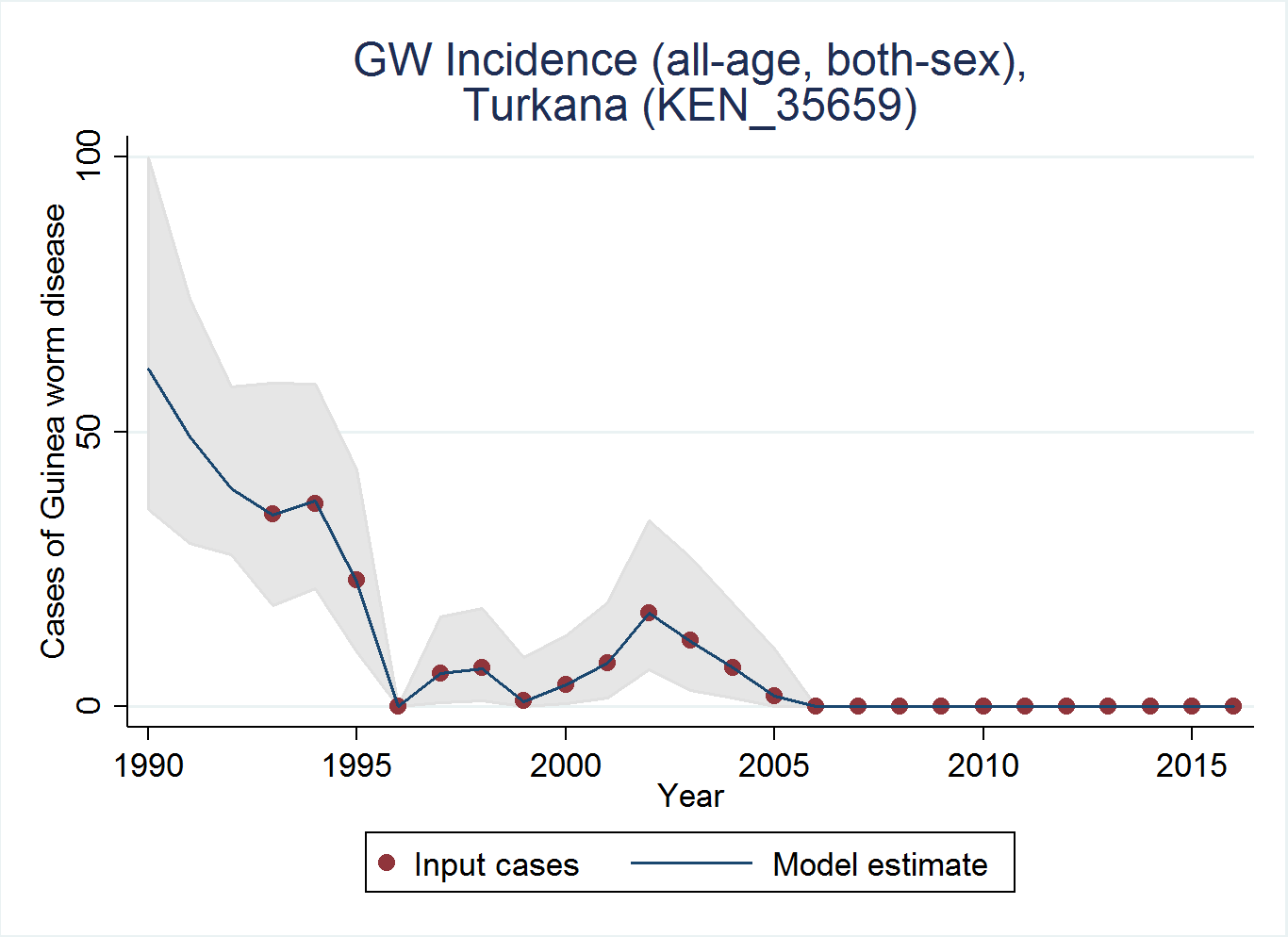


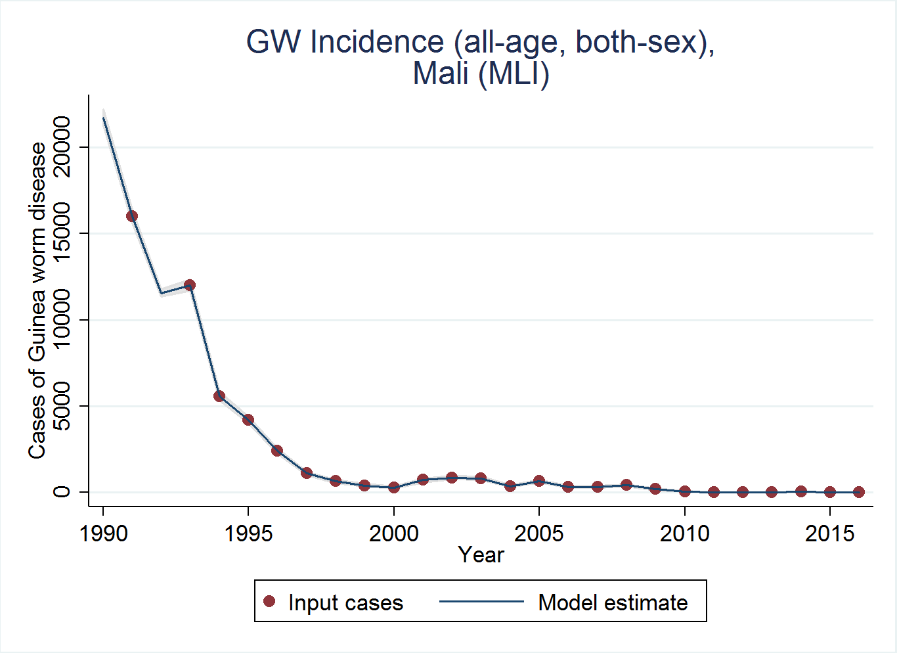

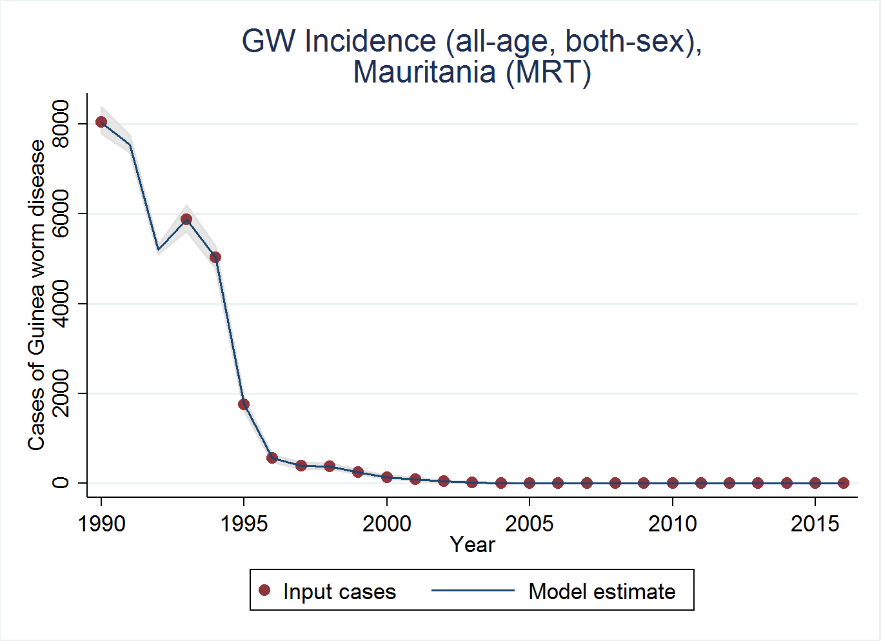


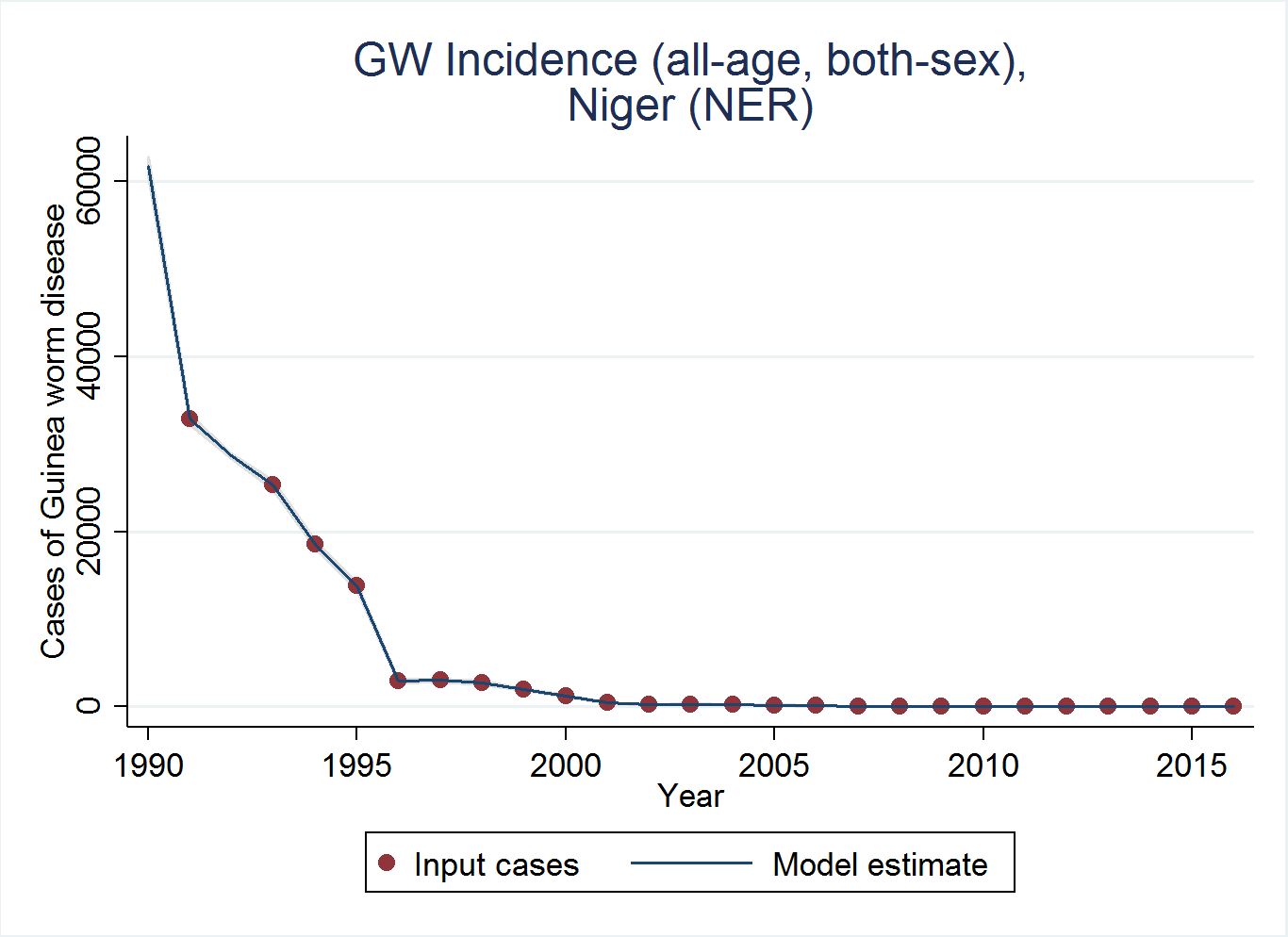

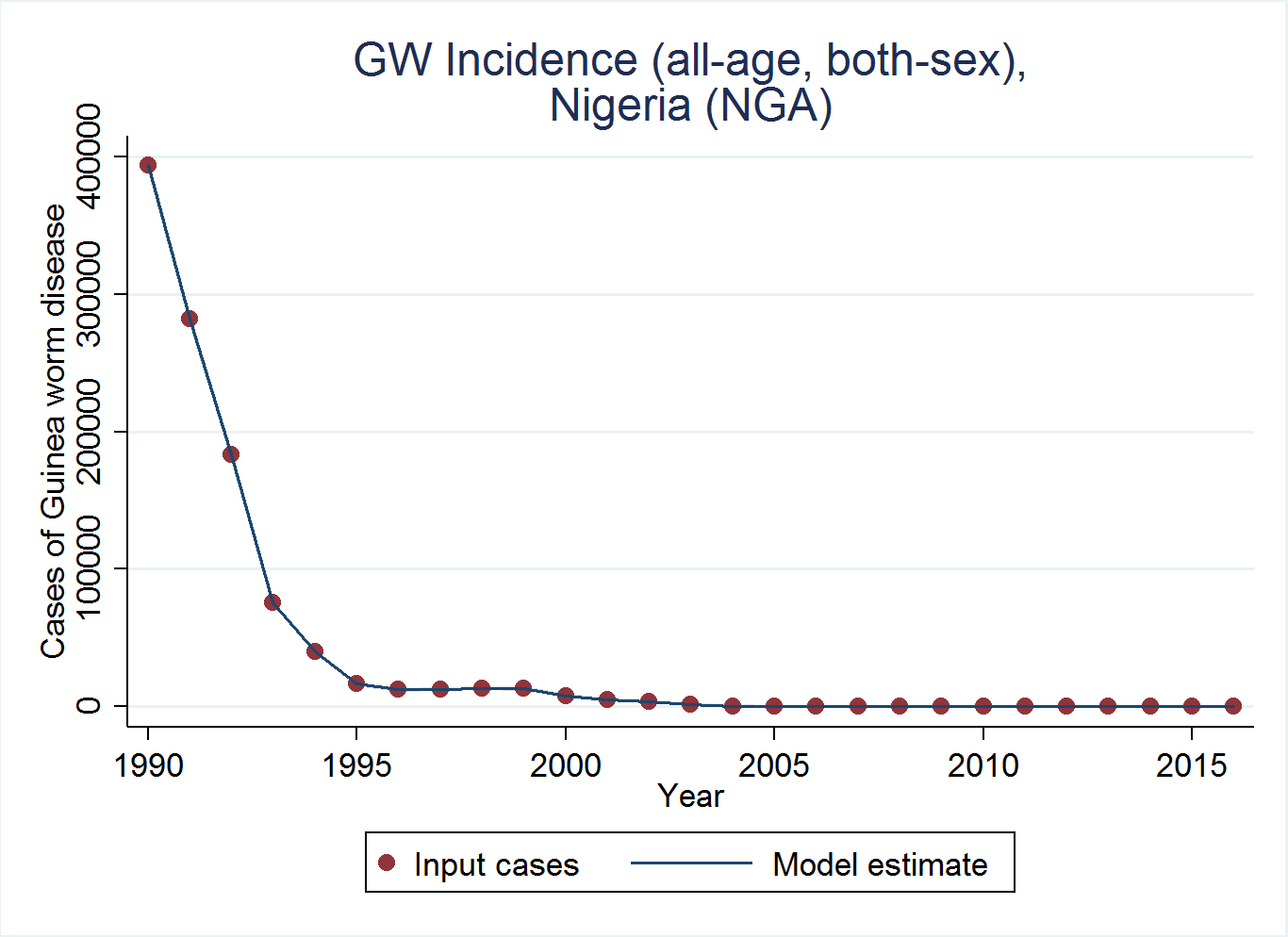

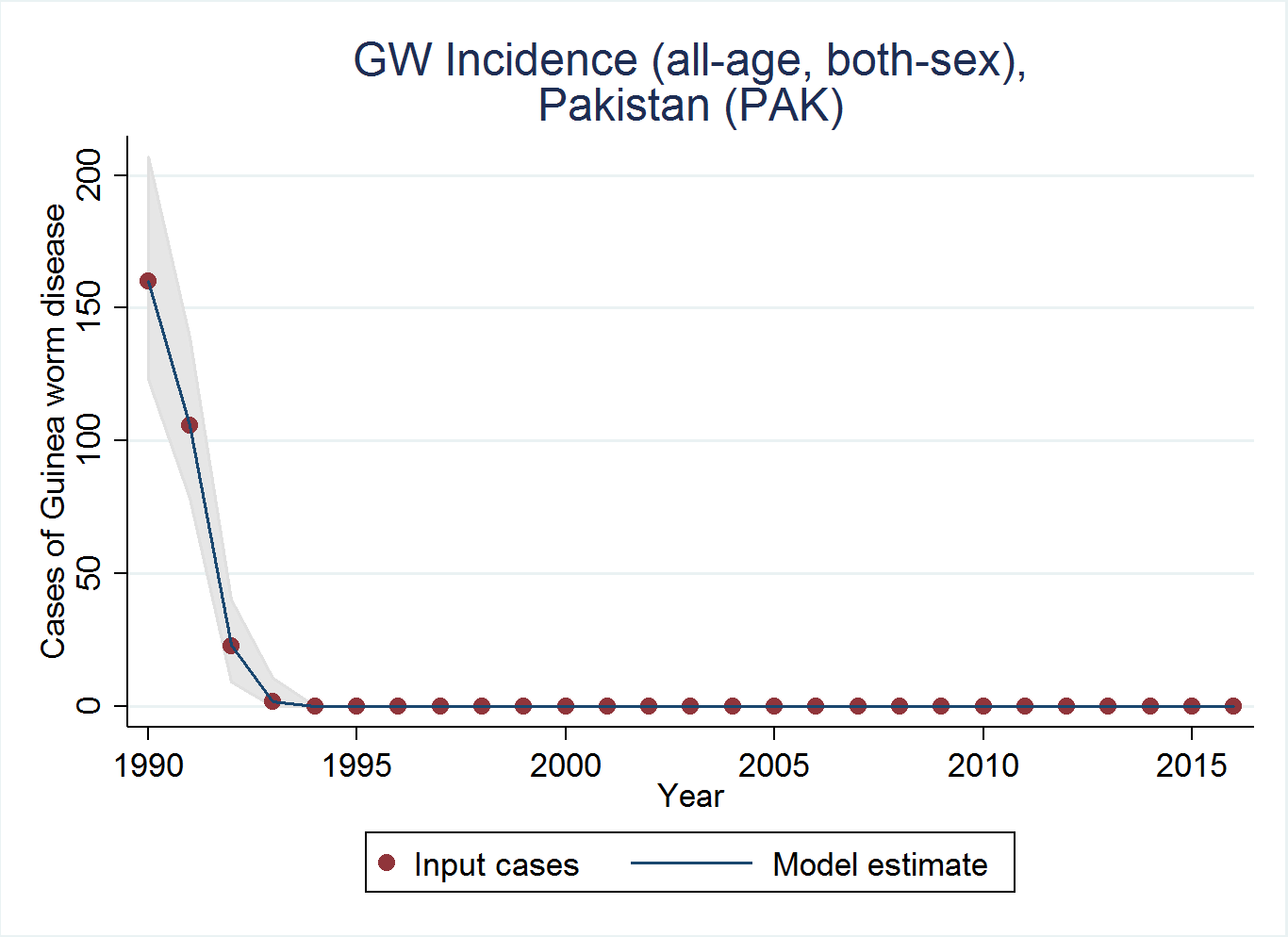

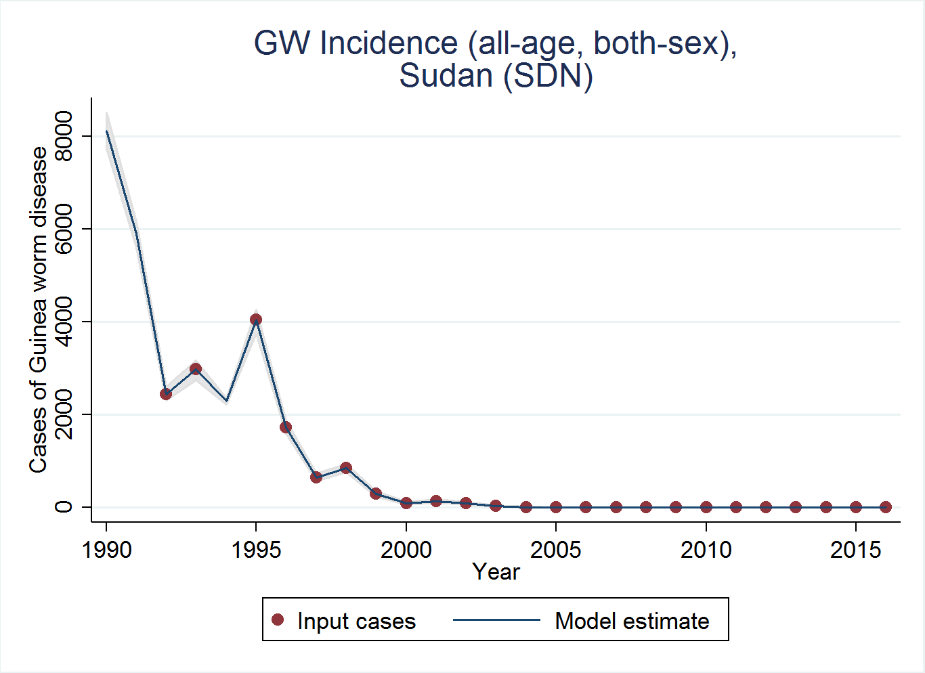

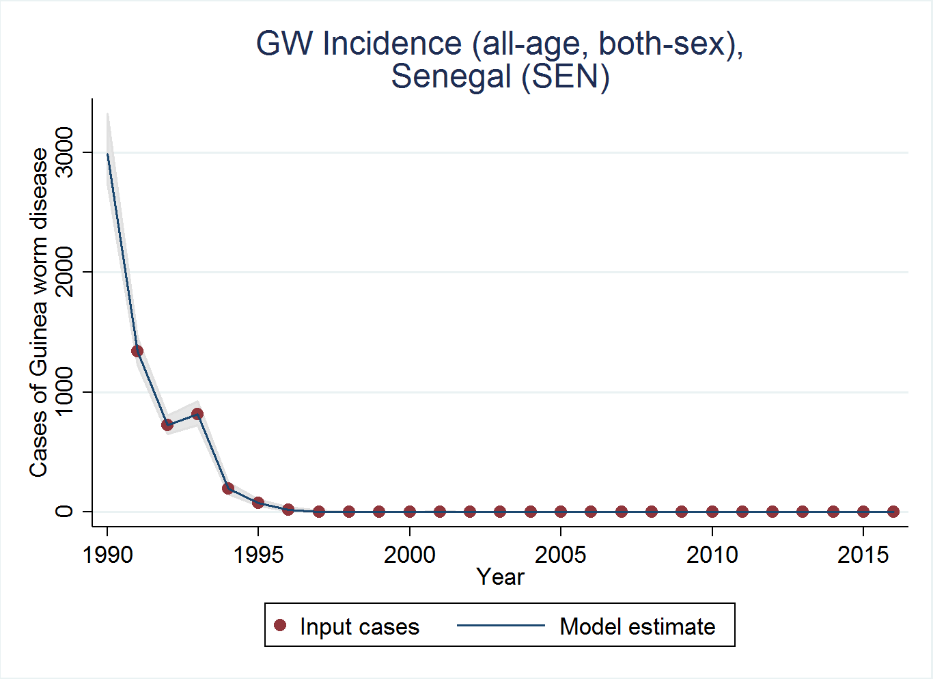

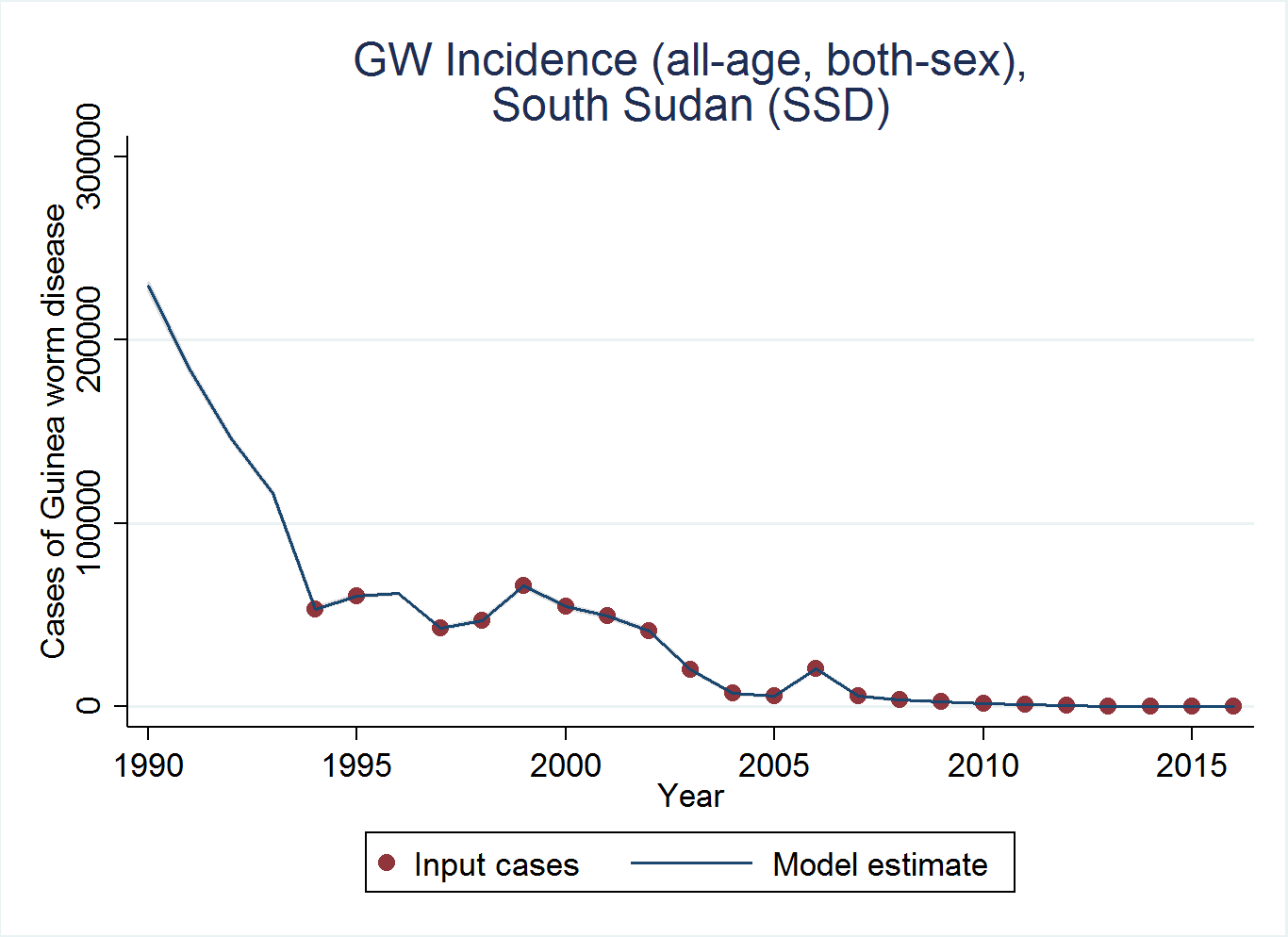

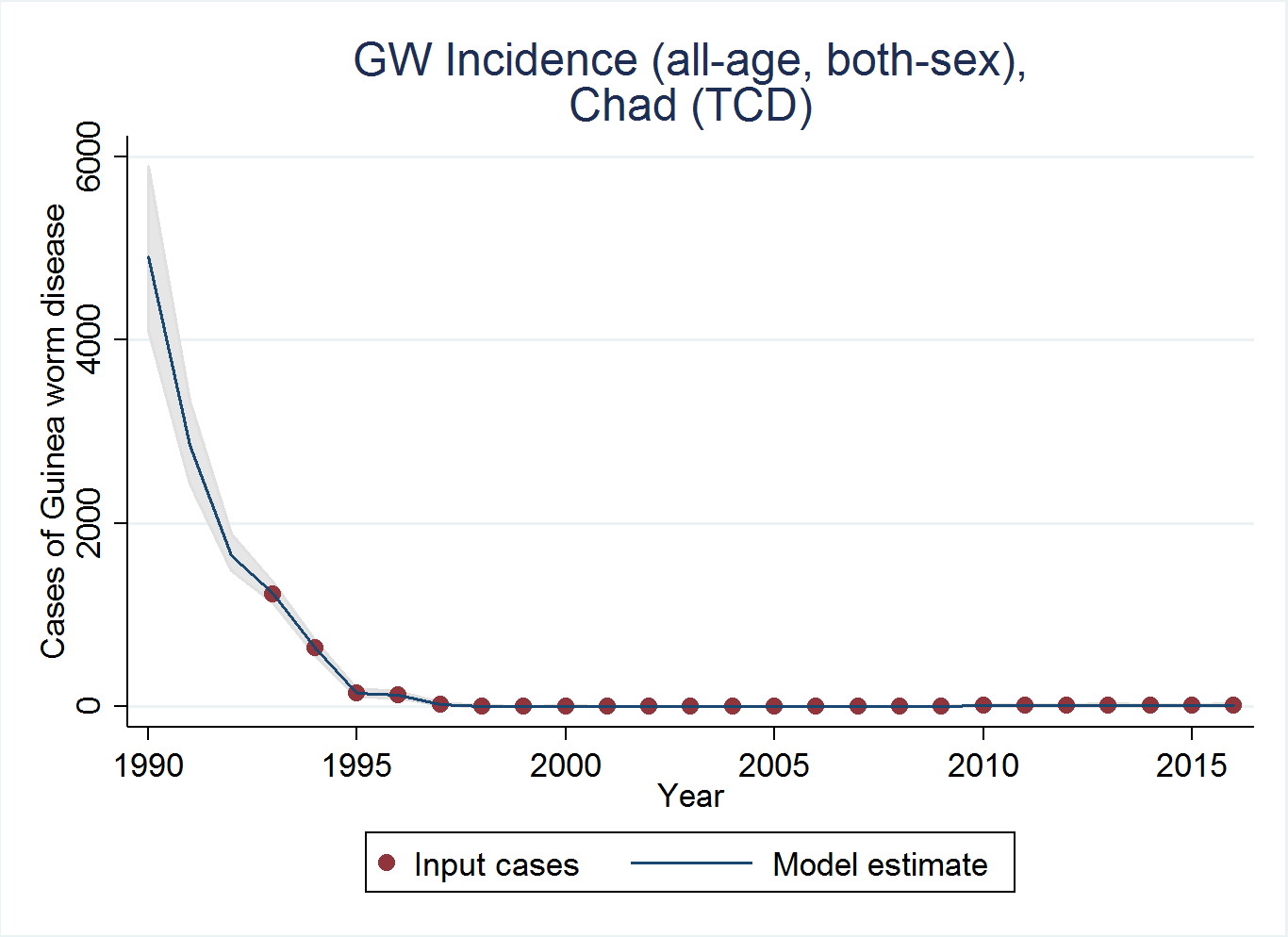

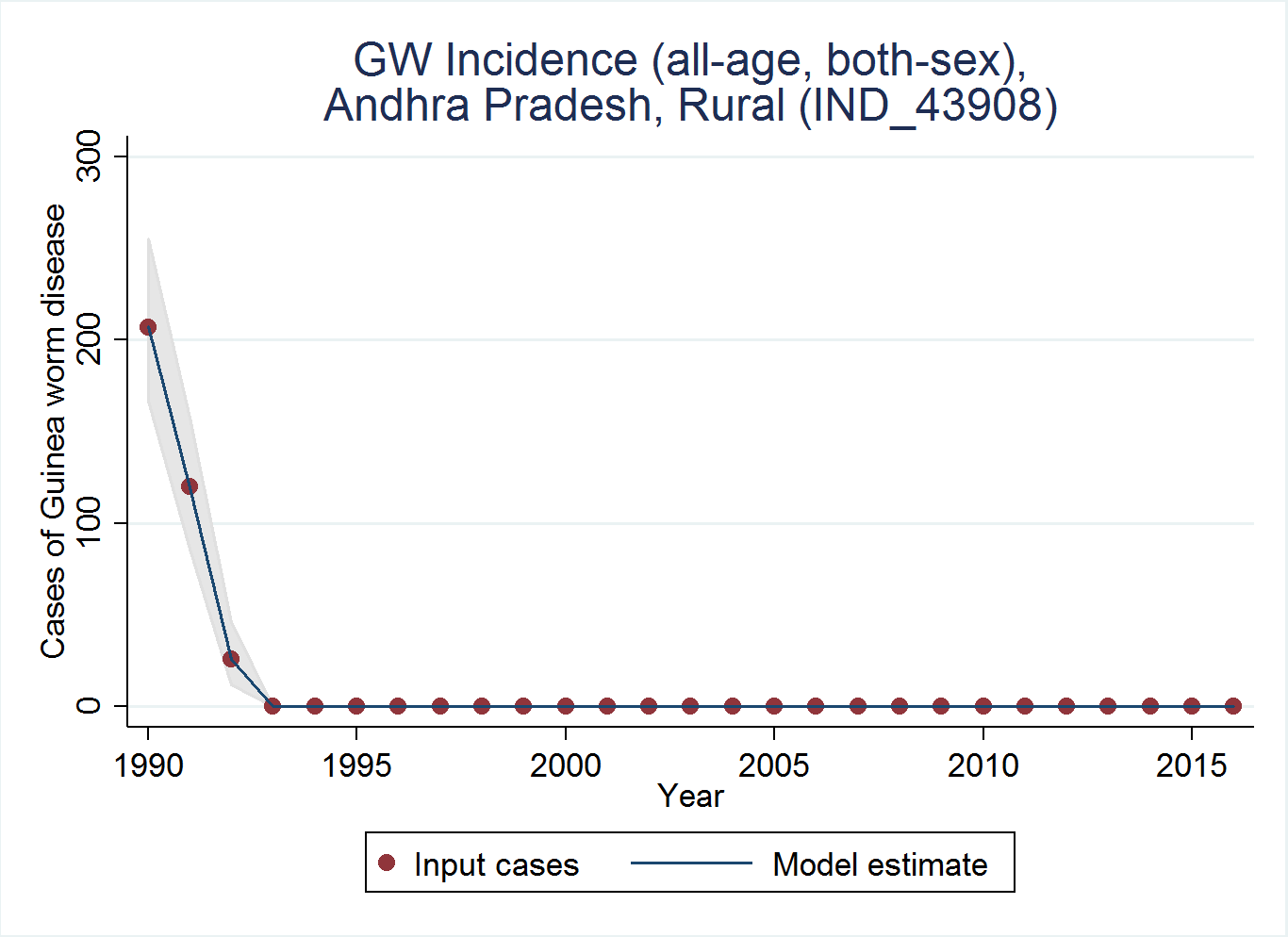

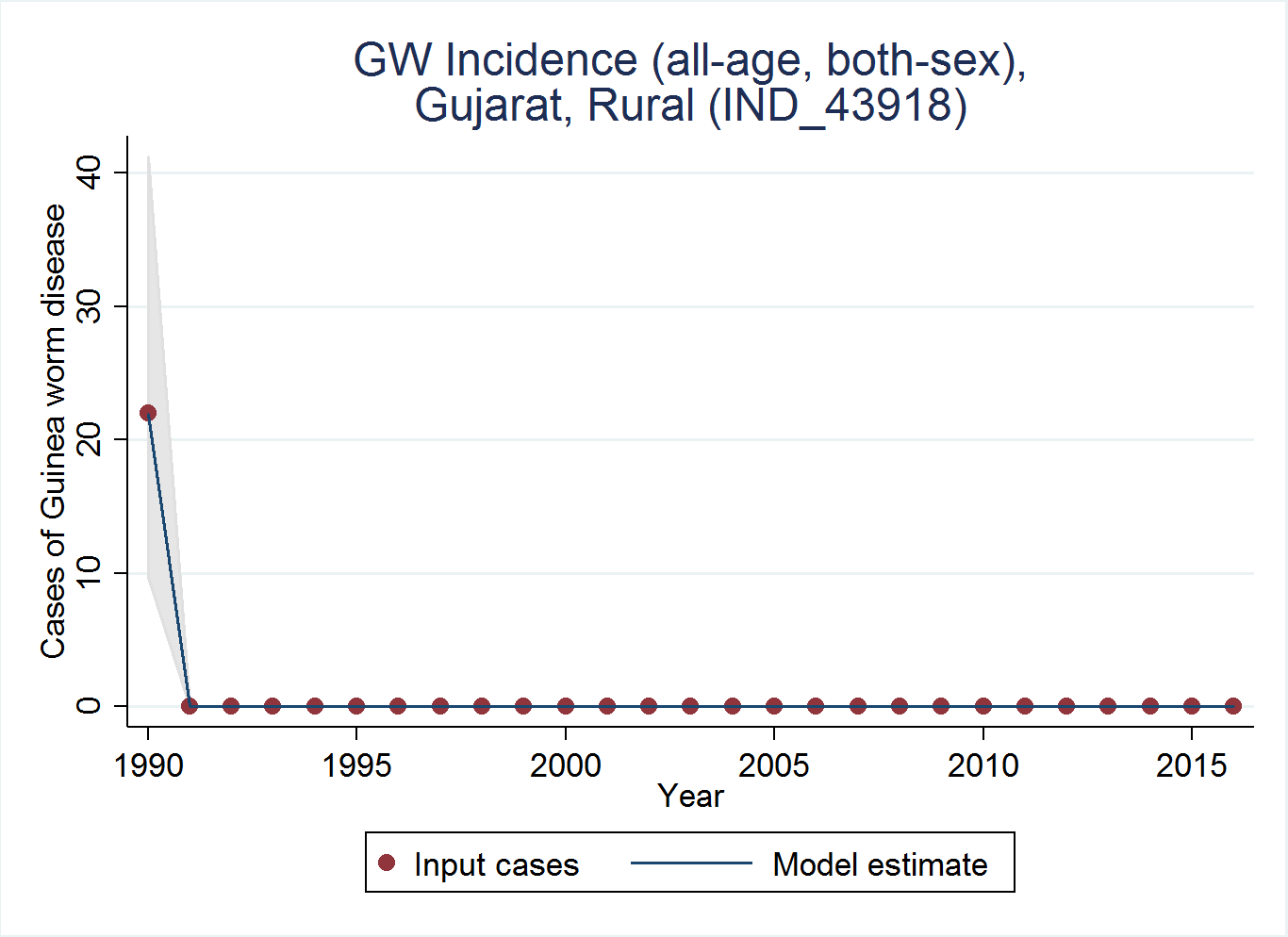

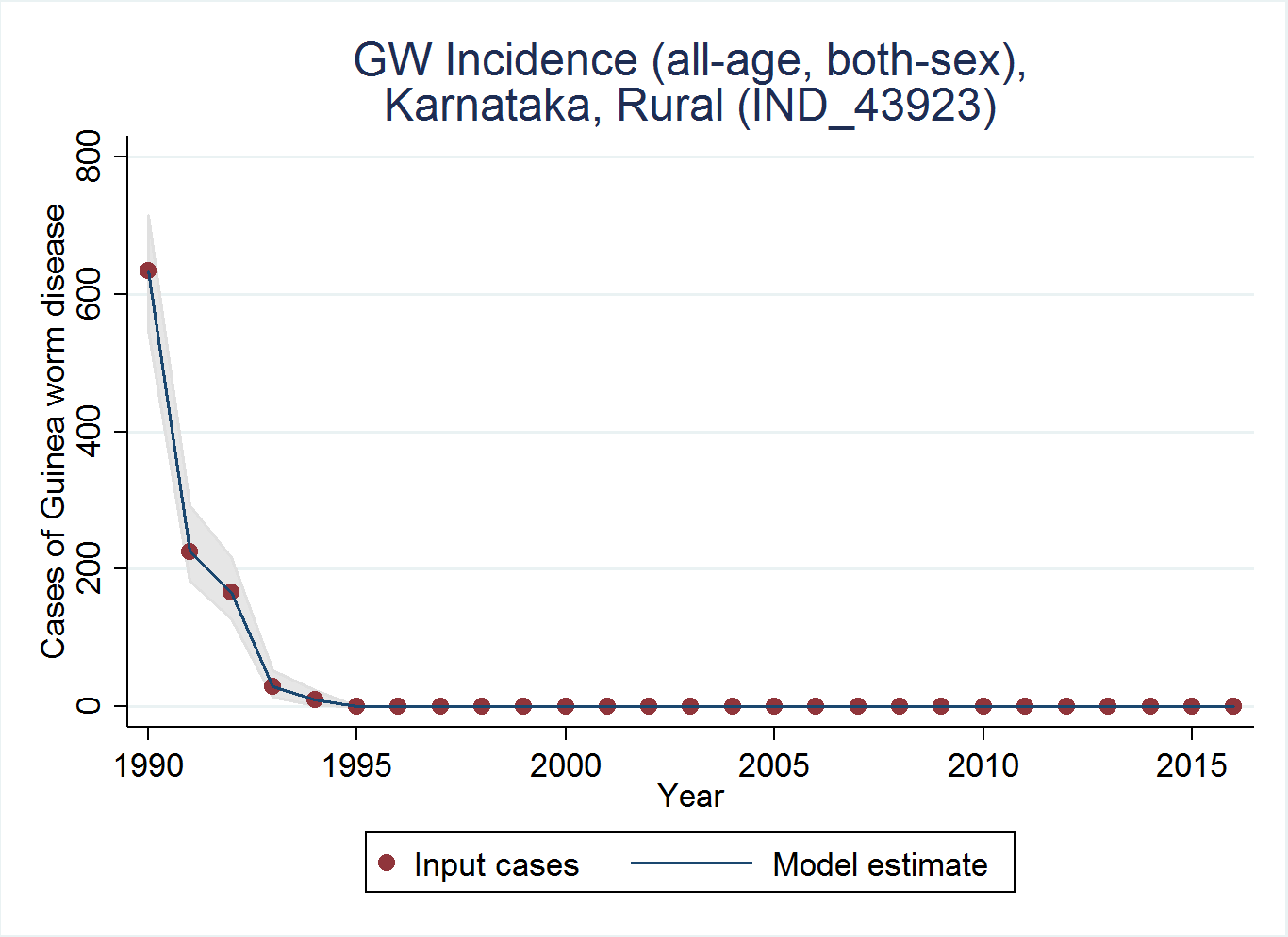

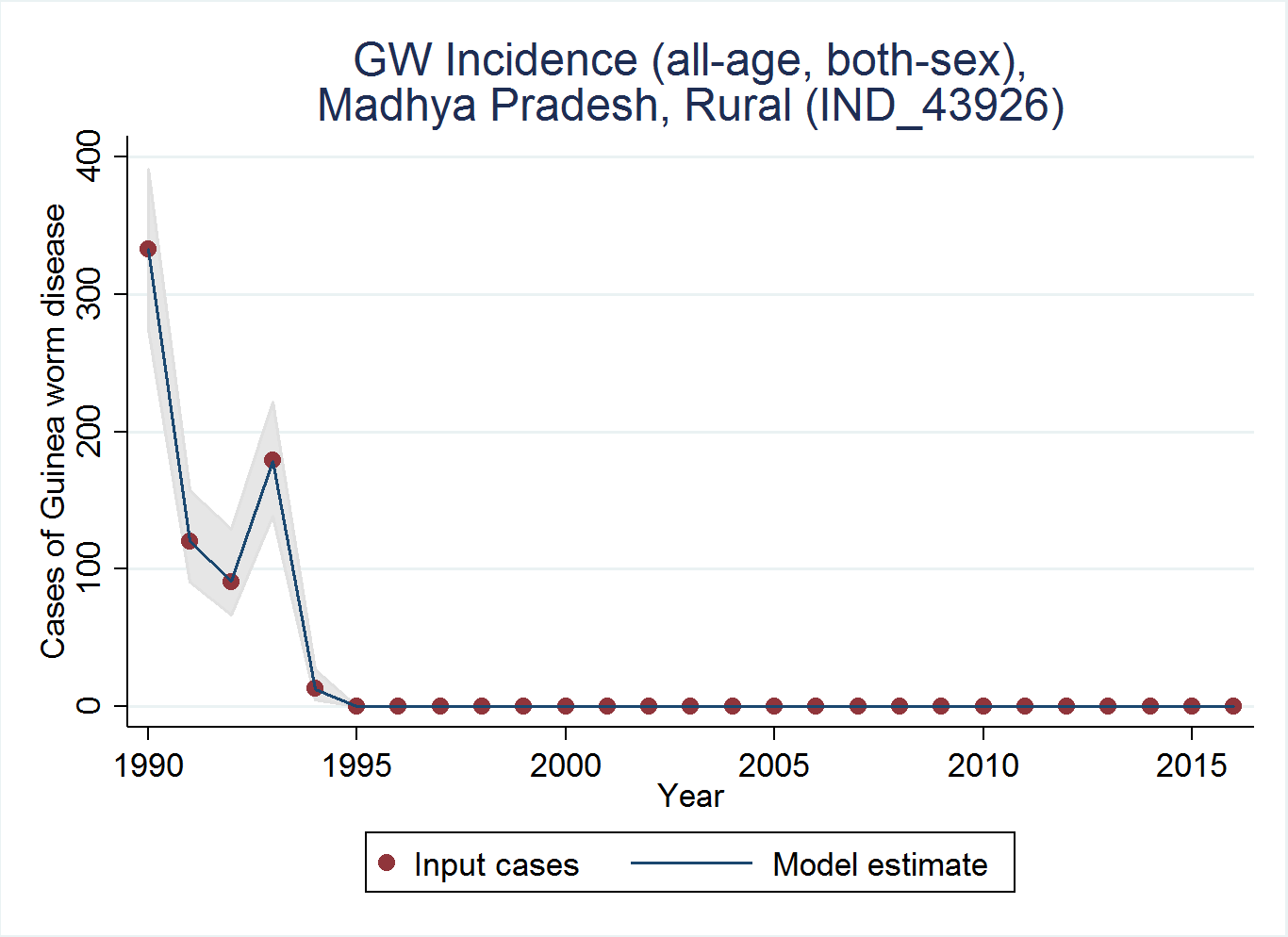

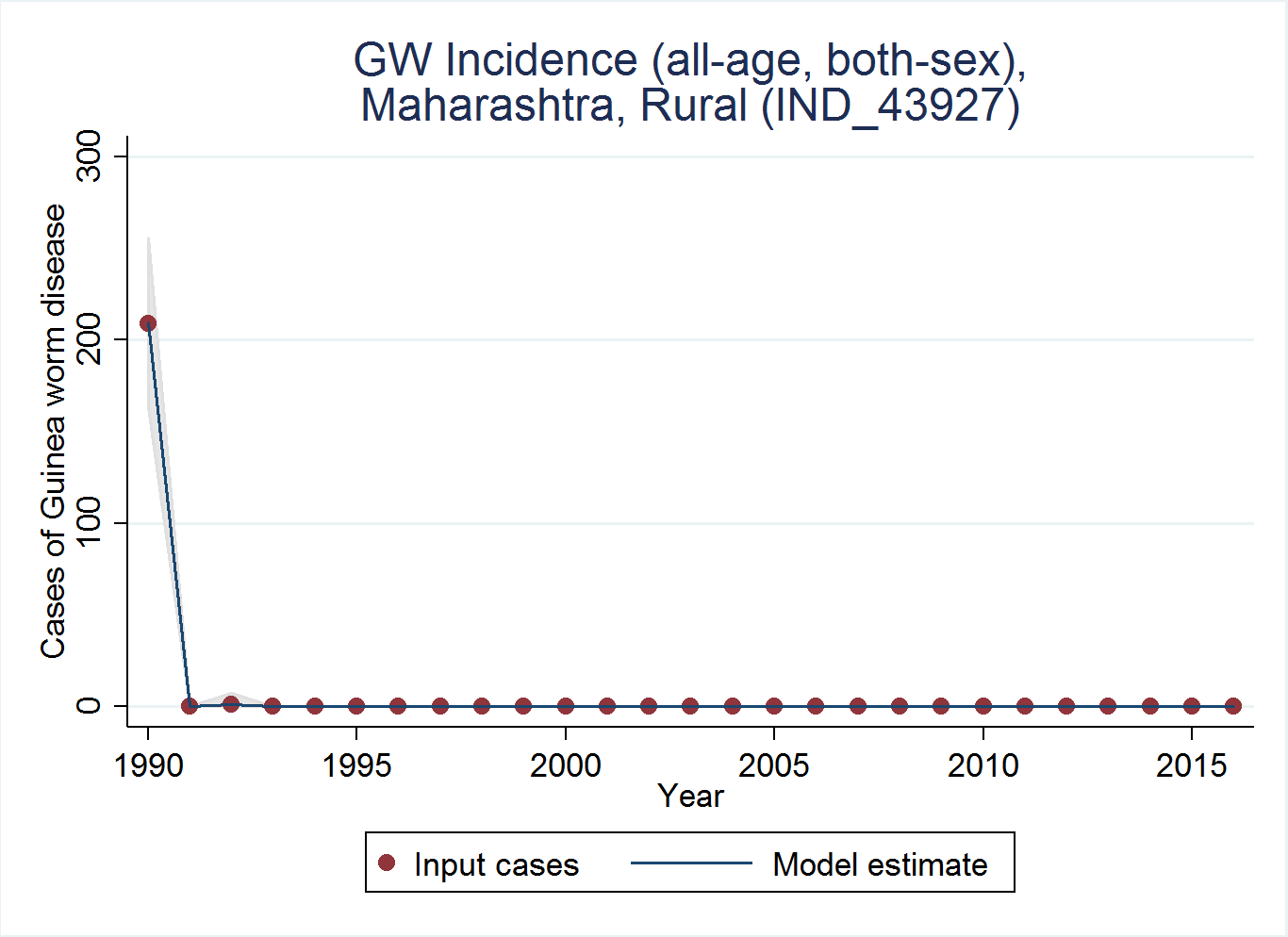

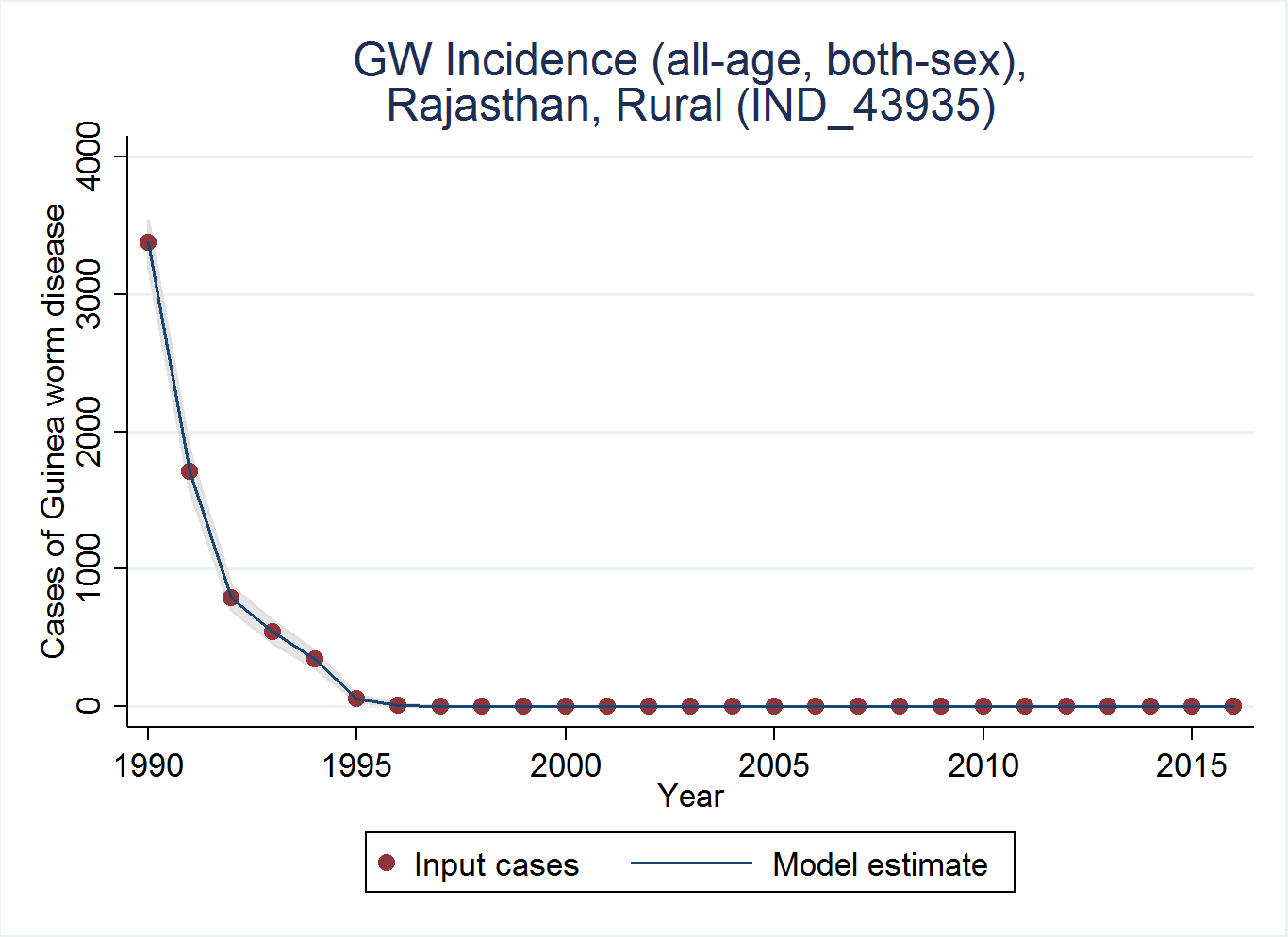

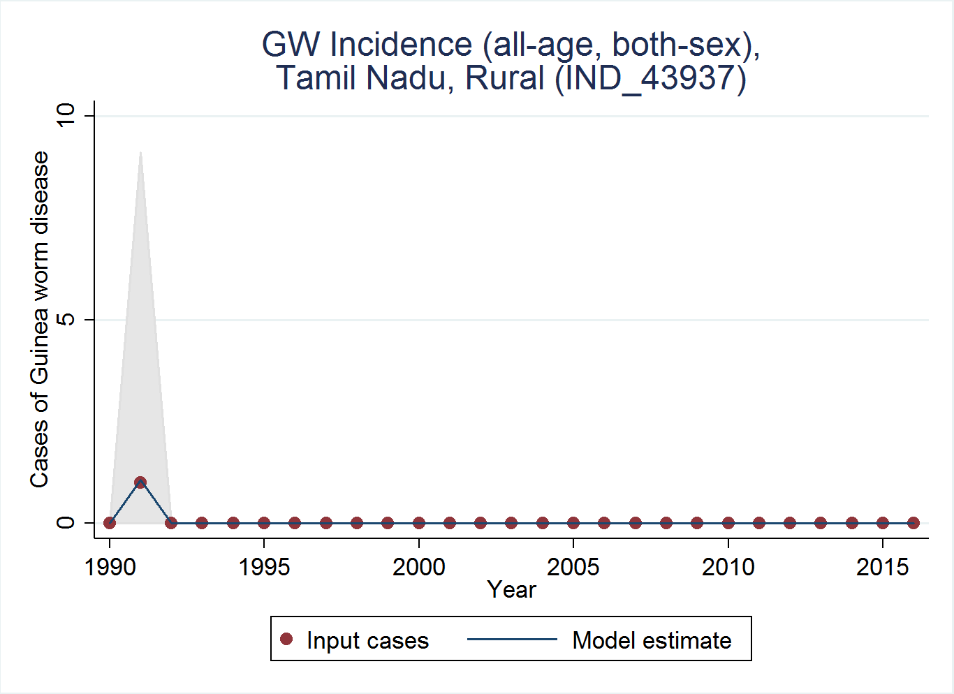

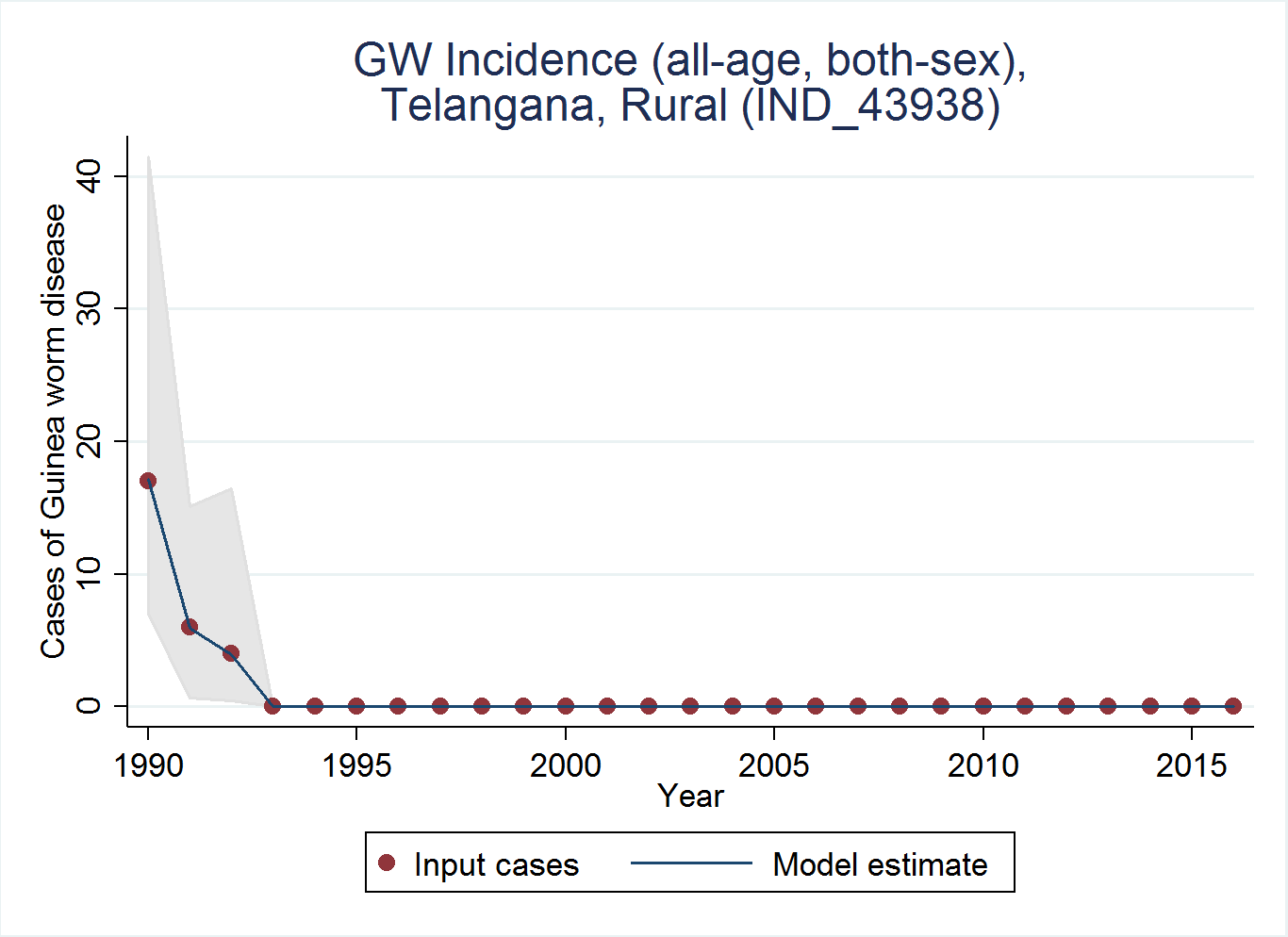

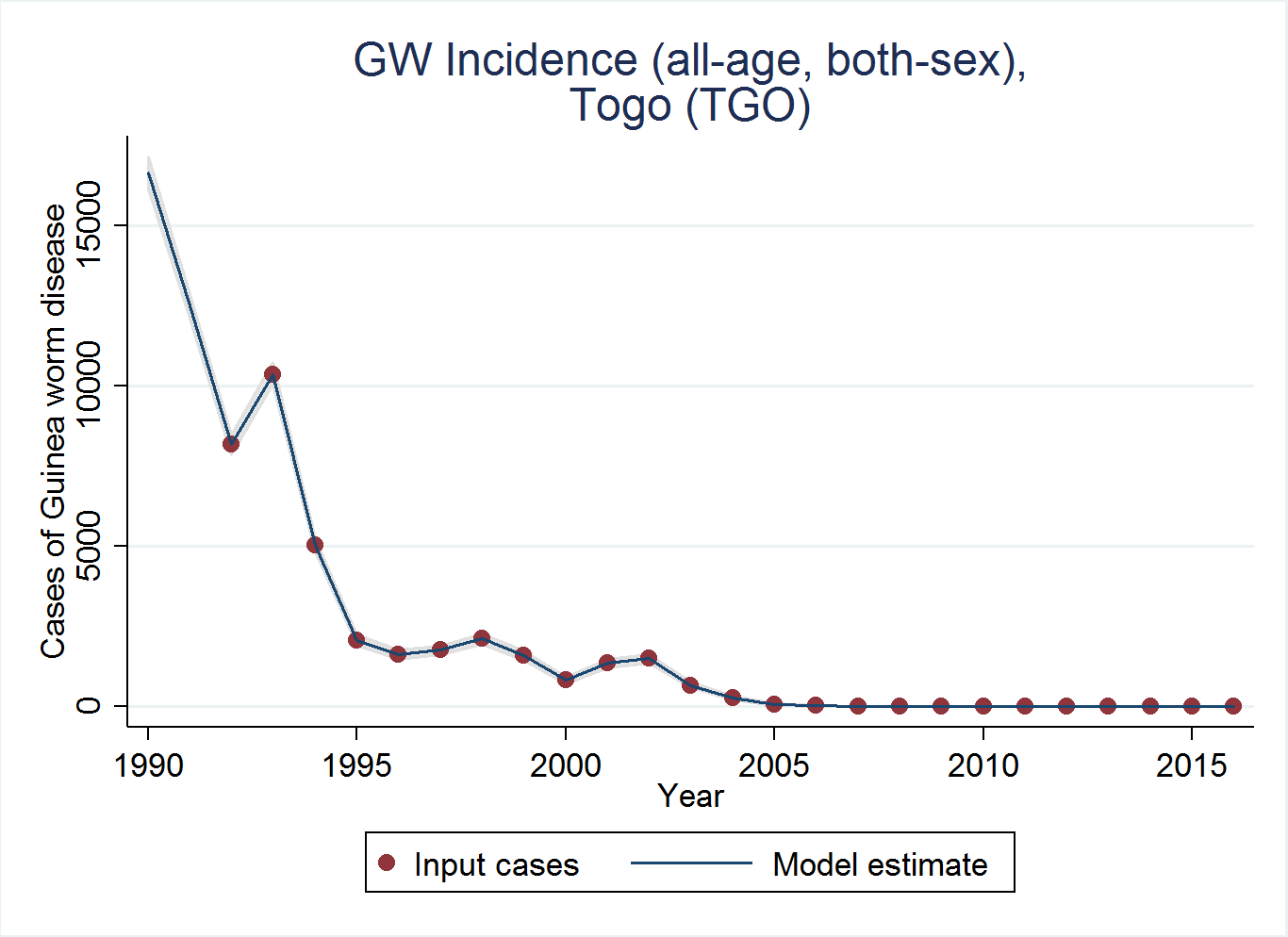

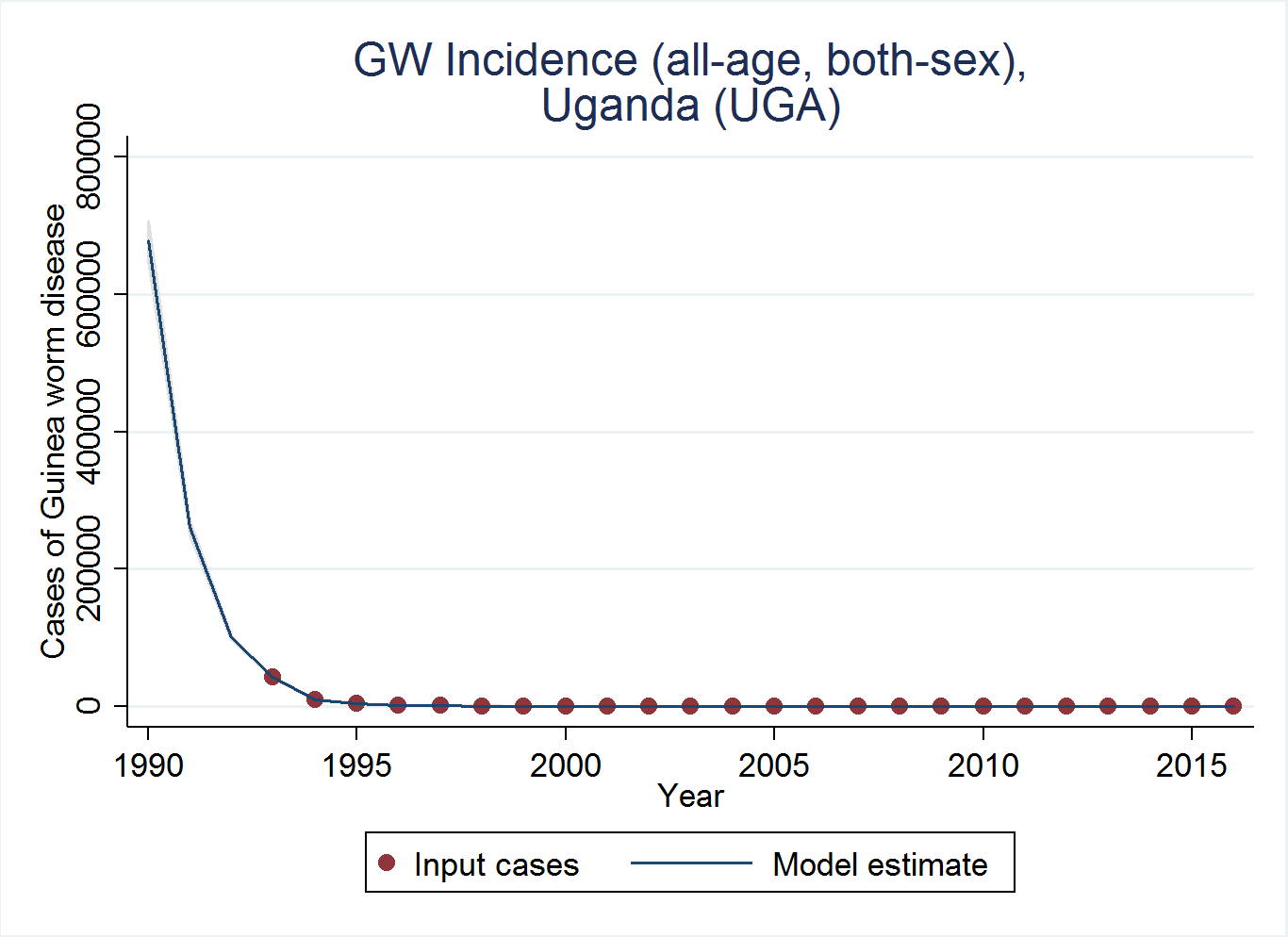

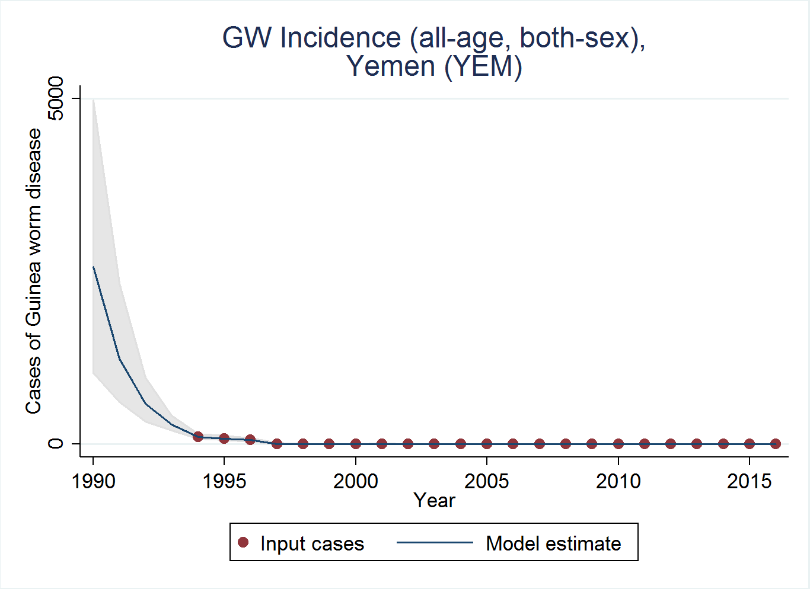


# References

1. Biswas G, Sankara DP, Agua-Agum J, Maiga A. Dracunculiasis (guinea worm disease): eradication without a drug or a vaccine. Philos Trans R Soc Lond B Biol Sci 2013;368:20120146.

2. Richards F, Hopkins D. Surveillance: the foundation for control and elimination of dracunculiasis in Africa. Int J Epidemiol 1989;18:934-43.

3. Hopkins DR, Ruiz-Tiben E, Weiss A, Withers PC, Jr., Eberhard ML, Roy SL. Dracunculiasis eradication: and now, South Sudan. Am J Trop Med Hyg 2013;89:5-10.

4. Hopkins DR, Ruiz-Tiben E, Ruebush TK, Diallo N, Agle A, Withers PC, Jr. Dracunculiasis eradication: delayed, not denied. Am J Trop Med Hyg 2000;62:163-8.

5. Hopkins DR, Ruiz-Tiben E. Strategies for dracunculiasis eradication. Bull World Health Organ 1991;69:533-40.

6. Hopkins DR. Disease eradication. N Engl J Med 2013;368:54-63.

7. Hopkins DR. The Guinea worm eradication effort: lessons for the future. Emerg Infect Dis 1998;4:414-5.

8. Dracunculiasis. Wkly Epidemiol Rec 1996;71:141-8.

9. WHO Collaborating Center for Research TaEoD. Guinea Worm Wrap-Up. Atlanta, GA: Centers for Disease Control and Prevention; 1991 December 31, 1991.

10. WHO Collaborating Center for Research TaEoD. Guinea Worm Wrap-Up. Atlanta, GA: Centers for Disease Control and Prevention; 1990 December 31, 1990.

11. WHO Collaborating Center for Research TaEoD. Guinea Worm Wrap-Up. Atlanta, GA: Centers for Disease Control and Prevention; 1994 April 20, 1994.

12. WHO Collaborating Center for Research TaEoD. Guinea Worm Wrap-Up. Atlanta, GA: Centers for Disease Control and Prevention; 1993 October 18, 1993.

13. WHO Collaborating Center for Research TaEoD. Guinea Worm Wrap-Up. Atlanta, GA: Centers for Disease Control and Prevention; 1990 April 15, 1990.

14. WHO Collaborating Center for Research TaEoD. Guinea Worm Wrap-Up. Atlanta, GA: Centers for Disease Control and Prevention; 1994 December 30, 1994.

15. WHO Collaborating Center for Research TaEoD. Guinea Worm Wrap-Up. Atlanta, GA: Centers for Disease Control and Prevention; 1993 January 20, 1993.

16. WHO Collaborating Center for Research TaEoD. Guinea Worm Wrap-Up. Atlanta, GA: Centers for Disease Control and Prevention; 1989 April 15, 1989.

17. WHO Collaborating Center for Research TaEoD. Guinea Worm Wrap-Up. Atlanta, GA: Centers for Disease Control and Prevention; 1992 September 18, 1992.

18. WHO Collaborating Center for Research TaEoD. Guinea Worm Wrap-Up. Atlanta, GA: Centers for Disease Control and Prevention; 1995 July 14, 1995.
