# Supplementary material for: Slaying little dragons: the impact of the Guinea Worm Eradication Program on dracunculiasis disability averted from 1990 to 2016
Source: Gates Open Res. 2018 Jun 18;2:30. [Version 1] doi: 10.12688/gatesopenres.12827.1 (PMC6139381; doi:10.12688/gatesopenres.12827.1)
Supplement: Peer review history

# Peer review: Referee response for version 1

**DOI:** 10.21956/gatesopenres.13900.r26527

**Published:** 2018-07-13

**Authors:** Philip Downs

**Affiliations:** 1 Sightsavers, Durham, NC, USA

Cromwell et al. establish an important metric to assess the impact of the Guinea worm eradication programme and compare other disease and elimination programmes.  As noted by the authors, the model presented may under represent the impact on burden as earlier prevalence estimates are likely under reported; estimates prior to 1990 put the prevalence of GWD closer to 3.5 million cases.  A major limitation is not accounting for the quantity of worm burden per case and accounting for the mental health burden of the disease as part of sequelae.  Article highlights the importance of all elimination programmes to capture and publish parameters of disease sequelae as soon as possible. 

 Potential typo in one of the sentences - I believe the authors intended to mean unpublished in the sentence "Our analysis includes a review of the scientific literature on GWD, which is sparse and largely published before 1990."

I have read this submission. I believe that I have an appropriate level of expertise to confirm that it is of an acceptable scientific standard.

# Response

**Published:** 2018-07-24

**Authors:** Simon Hay

Thank you for the comment. We agree there is likely additional morbidity associated with the number of worms per individual infected. Depending on the availability of data, it may be possible to update the model in the GBD with an estimate of worm burden as it relates to either degree of pain and limited mobility or duration of disease. Mental health outcomes are difficult to estimate in this framework as data on morbidity due to Guinea worm disease likely come from cross-sectional studies. In addition to attributing disability weights to mental health states, there is not enough evidence to assign a duration to mental health outcomes associated with Guinea worm disease to include in the calculation of a DALY.

# Peer review: Referee response for version 1

**DOI:** 10.21956/gatesopenres.13900.r26532

**Published:** 2018-07-05

**Authors:** Guillaume Chabot-Couture

**Affiliations:** 1 Institute for Disease Modeling, Bellevue, WA, USA

Good manuscript overall, and a useful addition to the GW literature.

 Under-reporting is discussed as an important limitation, and effort is make to evaluate how much cases could have been under reported when it comes to estimating impact of GWEP.

 Good review of information on sequelae. As they discuss, multiple worms could cause more sequelae by hitting the same patient. Patients with multiple worms could have been under-reported, and multiple worms could have been more frequent at the beginning of the GWEP which could make the quoted results an under-estimate of the impact on burden. This should be explicitly modeled as part of their sensitivity analysis.

 In Chad, from 2000 to 2010, there must have been cases, but surveillance was missing them. It would be more accurate to interpolate a burden of 10-20 cases during those years than to take the zero reports as is. Alternatively, this could be reported as "*" as it is done in the appendix.

 A note should be made about DRC and how the absence of reporting from that country without good surveillance present may be masking some GW burden.

I have read this submission. I believe that I have an appropriate level of expertise to confirm that it is of an acceptable scientific standard.

# Response

**Published:** 2018-07-24

**Authors:** Simon Hay

We thank the reviewer for the comment. As we mention above, we will review data sources to determine if worm burden can be included in future iterations of the model. While the point about DRC is noted, we would need additional data sources from the Ministry of Health to re-consider the geographic exclusions used in the analysis.  The suggestion regarding Chad can be tested in a subsequent iteration of the GBD.

# Peer review: Referee response for version 1

**DOI:** 10.21956/gatesopenres.13900.r26528

**Published:** 2018-06-29

**Authors:** Teshome Gebre

**Affiliations:** 1 International Trachoma Initiative, Addis Ababa, Ethiopia

The article is very well written and the data presented on the burden of GWD accurately reflects the situation in the known endemic countries to the best of my knowledge.  Most of the global program data before 1990 was mainly based on estimates from national health facility morbidity statistics or commonly known as health facility reports.  Obviously, these data sources have their own limitations as GWD or Dracunculiasis could be misdiagnosed or misclassified during recording and reporting by health workers or statisticians.  The possibility of underreporting and overreporting exists at all levels of the health care delivery nearly everywhere.  On the other hand, since GW is a disease of poverty affecting the poorest of the poor in very remote and underserved settings, it is highly likely that there could have been plenty of cases missed or uncaptured by the health system due to lack access to the health service and hence undermining the reported incidence/prevalence.  I have noticed that the GBD estimates have taken this scenario into account while calculating the annual incidence for each country.  On the other hand, most of the GWD burden reported after 1990 and most importantly after 1995 are likely to reflect the precise picture of the GW situation in most of the endemic countries since there was a village-based surveillance system established in nearly all countries where there was no civil strife occurring like in the case of Sudan.  I am in agreement with the way the DALYs were calculated.  Most importantly, it is good that the authors considered the GWD burden for the period 1990 - 2016.  It would have been an over-estimate  had the authors considered the GWD burden for the period 1986 - 2016 to calculate the DALYs averted by the global program.  I am hesitant to attribute all the 80% GW case reduction observed during 1990 - 1995 to those soft interventions like filter distribution, health education and Abate application.  I would say that the reduction was mainly achieved due to improved reporting as a result of instituting trained village based volunteers and thereby establishing effective village based surveillance system with robust supervisory network from national level all the way down to district or county or LGA levels as the case may be in each endemic country.  Overall, I am appreciative of the work done by the authors.  I have seen firsthand the immense suffering and terrible consequences of this crippling disease and it is indeed worth publishing this work to celebrate the great breakthrough attained in eradicating this dreadful disease.

I have read this submission. I believe that I have an appropriate level of expertise to confirm that it is of an acceptable scientific standard.

# Response

**Published:** 2018-07-24

**Authors:** Simon Hay

Thank you for the comments. We agree with the reviewer that reductions in cases post from 1990 to 1995 may reflect changes in reporting procedures.  The results do include larger uncertainty intervals for the 1990 estimates compared to later time points, largely driven by the model attempting to correct for under-reporting or missing data from the early 1990s.
